# Prevalence and factors associated with substance use among street children in Jimma town, Oromiya national regional state, Ethiopia: a community based cross-sectional study

**DOI:** 10.1186/s13011-020-00304-3

**Published:** 2020-08-20

**Authors:** Mengistu Ayenew, Teshome Kabeta, Kifle Woldemichael

**Affiliations:** 1 Department of Public Health, College of Medicine and Health Science, Mizan Tepi University, Mizan Aman, Ethiopia; 2 Department of Epidemiology, Faculty of Public Health, Jimma University, Jimma, Ethiopia

**Keywords:** Substance use, Street children, Cigarette smoking, Khat chewing, Drinking alcohol

## Abstract

**Background:**

Street children constitute a marginalized population in most urban centers of the world. According to UN sources, there are up to 150 million street children in the world today. The estimated number of children who live on the streets in Ethiopia was 150,000, of which about 60,000 of them in Addis Ababa. However, aid agencies estimate that the problem may be far more serious, with nearly 600,000 street children country-wide and over 100,000 in Addis Ababa. World Health Organization estimates that globally, 25–90% of street children indulge in substance use. Even if substance use has become a common problem in Ethiopia, most of the studies done mainly focused among schools, college and university students. Research on street children and their substance use habits in Ethiopia was limited and specifically non in Jimma town.

**Objectives:**

To assess the prevalence and factors associated with substance use among street children in Jimma town of Ethiopia in 2019.

**Methods:**

Cross sectional study was undertaken from March 1–31, 2019. Complete enumeration of study subjects was done and all 312 children of the streets were included. Interviewer administered structured questionnaires was used to collect the data. Bivariable logistic regression was carried out to select candidate for multiple logistic regression analysis with *p*-value < 0.25 at 95% confidence. Multiple logistic regression was carried out with those candidate variables using backward method and the associations predictors to the response variable was declared with *p* value of < 0.05 at 95% confidence level.

**Result:**

Three hundred twelve street children were included in the study. The prevalence of substance use was 30.8% with 95% CI [25–36.2]. Age > 14 [AOR: 1.97 95%CI:1.00–3.889], attending grade 1-4th [AOR: 0.33 95%CI:0.151–0.737], attending 5th grade and above [AOR: 0.27 CI:0.093–0.756], child whose mother used substances [AOR: 7.78 95%CI:3.00–20.11], child did not know his maternal substance use status [AOR:5.1 95%CI: 2.19–11.81], child whose sibling use substance [AOR: 2.23 95%CI:1.254–5.63], best friend substance use [AOR: 11.01 95%CI:5.47–25.04] and staying 12–60 months on the street [AOR:3.00 95%CI:1.511–5.96] and staying > 5 years on the street [AOR:4.6 95%CI:1.06–19.7] were significantly associated with substance use.

**Conclusion and recommendation:**

The prevalence of substance use among street children in Jimma town was high. Mothers and siblings have crucial roles in determining substance use behavior of the children. Stakeholders who are working on the improvement in the life of children of the street should try to satisfy the need of the children by intervening at individual level, at family or community level and at levels beyond community to lessen the problem to some extent. Researchers should do similar researches in more detail on these vulnerable but neglected groups of children to fully understand about the problem so the findings will be used as inputs for concerned bodies.

## Background

The history of substance utilization is as old as mankind. People have always had a desire to eat or drink substances because of their cultural practices, for relaxing, stimulating or euphoric [1].

The phrase ‘street children’ has been used to refer to a population of youngsters either alone or in groups, perform informal activities such as doing odd jobs, begging, wandering, and other activities necessary for themselves or their family’s survival [2]. Street child is defined as any girl or boy, for whom the street has become his or her habitual abode and/or source of livelihood; and who is inadequately protected, supervised, or directed by responsible adults [3].

The nature of continuous exposure to the street and its associated lifestyles makes street children vulnerable to the use of psychoactive substances. Street children’s drug use often commences with alcohol, tobacco and inhalants which are legal and easily accessible in most countries. World Health Organization (WHO) estimates that globally, 25–90% of street children indulge in substance use [4].

A study which was conducted in Accra, Ghana revealed that substance use was relatively high as 12 and 16.2% reported daily use of alcohol and marijuana respectively. There were age and sex differences in substance use among the sample. While alcohol use decrease with age, marijuana use on the other hand increases with age [5].

Street children have a greater burden than other poor children who are supervised by adults. The inherent dangers of being in the street situation, economic deprivation, lack of adult protection and inadequate socialization and, lack of protection make them extremely vulnerable. Many street children are involved in harmful use of psychoactive substances which can lead to increase the chance of accidents, violence, unprotected sex that leads to unwanted pregnancy [6].

According to the sources of United Nation (UN), there are up to 150 million street children in the world today. Chased from home by violence, drug and alcohol abuse, the death of a parent, family breakdown, war, natural disaster or simply socioeconomic collapse, many destitute children are forced to eke out a living on the streets, scavenging, begging, hawking in the slums and polluted cities of the developing world [7].

According to the Ethiopian Ministry of Labor and Social Affairs, 150,000 children live on the streets in Ethiopia from which about 60,000 of them in the capital Addis Ababa. However, aid agencies estimate that the problem may be far more serious, with nearly 600,000 street children country-wide and over 100,000 in Addis Ababa [8].

Related studies done in some Latin American countries identified the factors associated with substance use as parental drug use, lack of integration in school activities, challenges of children in education and changes to the traditional family structure, domestic violence and peer pressure [9, 10].

A study conducted in Brazil on drug use among street children and adolescents revealed that age, sex, duration on the street, and presence of family members had significant association with current substance use of street adolescent children [11].

Substance use among street children has been found to be influenced by factors such as gender, age, duration of homelessness and social networks (e.g. peer influence). Research has indicated that more males than females use alcohol, marijuana, cocaine and inhalants [12–14].

A meta-analysis done on the epidemiology of substance use among street children in resource-constrained settings showed that where the children stay at night, having family contact, duration on the street, old age, male sex had an association with substance use [15].

Other studies conducted in Mekele, and Guwahati City, Assam showed that age, sex, type of work, family substance use, educational status and duration on the street were factors for substance use among adolescent street children [16, 17].

Even if substance use has become a common problem in Ethiopia, most of the previously done studies were mainly on school, college and university students and research on street children and their substance use habits in Ethiopia, specifically in Jimma has been limited or none. So, this study aimed to assess the prevalence and factors associated with substance use among street children in Jimma town of Southwestern Ethiopia for better understanding of the problem in the country.

## Methods

### Study setting and design

A community based cross-sectional study was conducted in Jimma town, Oromiya National Regional State, Southwest Ethiopia from March 1–31, 2019. Jimma town is 352 km far from Addis Ababa, the capital city of Ethiopia. According to the 2015 National Urban System Study, the population of the city was 199,575 but the information regarding the number of street children in the town was not known.

### Sample size and sampling procedure

Complete enumeration of street children with age of 12–18 years old was done and a total of 365 street children were found in the town. Among these 37 children were stayed on the street for less than 1 month, 4 children had communication problem and 12 children were not available during the interview. After exclusion of all these children, finally 312 street children were included to the study.

A preliminary survey was conducted all over the urban kebeles of Jimma town with the help of community social workers and volunteers who were employees of Feyaa Integrated Development Organization. Feyaa Integrated Development Organization is one of Non-governmental organization found in Jimma town that works on supporting street children and orphans. Registration of all available street children in all corners of the town was made by recording of name, nick name, age, sex, friends’ name, usual area of residence, duration of stay on the street, anatomical deformity status and status of communication difficulties. For actual data collection, children were traced back based on the information collected from preliminary survey and transported to health center for interview.

### Data collection procedure and instrument

Data were collected using interviewer administered structured questionnaires by reviewing different published literatures and guidelines. The data were collected by five health officers, and the principal investigator supervised the whole data collection process. All data collectors took a one-day intensive training before data collection about objectives of the study, the issues of verbal assent and the rights of the respondents. In addition to data collectors, facilitators were also recruited and they were responsible for tracing the children based on the information collected by the preliminary survey and took them to health center where the data collection activities confidentially took place.

### Data quality assurance

Data collectors were trained on how to collect and handle data. The questionnaire prepared in English was translated into Amharic and was translated back to English to assess consistency and the Amharic version was used while carrying out the interview. Questionnaires were pretested on 5% of the street children in Agaro town prior to actual data collection. Some modifications and updating of tools were done based on the result of the pretest. Reviewing the filled questionnaires at the end of data collection every day for completeness, consistency and taking corrective measures were contentiously managed during the data collection periods.

### Study variables

The dependent variable was substance use whereas the independent variables were socio demographic factors (age, sex, religion, ethnicity, educational status, place of birth, daily income and type of work), personal factors (duration on the street, and sleeping place), environmental factors (availability of substances, accessibility of substances and affordability of substances) and family factors (parental status, maternal substance use, maternal job, maternal education, paternal substance use, paternal education, paternal job and sibling substance use).

### Data analysis procedures

Data were entered with Epi data version 3.1 and exported to SPSS version 23 for analysis. Data explorations were done to examine different characteristics of the data. After cleaning data, descriptive statistics like frequencies were computed for the categorical variables while, measures of central tendency and dispersion were summarized for continuous data. Bivariable logistic regression was carried out to select candidate for multivariable logistic regression analysis with *p*-value < 0.25 at 95% confidence. Then, candidate variables were entered to multiple logistic regressions model using backward elimination method. The degree of association was assessed using odds ratio and statistical significance were declared at 95% of confidence level and *p*-value of less than 0.05. Hosmer & Lemeshow’s test as well as its significance status was checked to assess the fitness of the model.

### Operational definitions

**Substance-** substances are any non-medical chemicals (including Khat, cigarette, alcohol, shisha … etc.) that affects activity of brain that distorts and make it work artificially and induce temporary happiness.

**Street children**: in our study, children who work and/or sleep on the street and within aged of 12–18 years who stayed on the street for at least 1 month.

**Substance use** - using one or more substances (Khat, cigarette, alcohol, shisha …) to alter mood or behavior at least once in the last 30 days.

**Availability of substances** – if a substance is available within 1 km radius of respondent’s residence area.

**Affordability** – if a substance can be bought with only 5 ETB or less.

## Results

### Socio demographic characteristics of street children in Jimma town

Three hundred twelve street children were included in the study, of which, 90.1%were males. Respondents with age of 12–14 years constitute 61.2% and the median age of the respondents was 14 years with interquartile range (IQR) of 2 years. Seventy-two (72.1%) of the children were born in rural area and most were Muslim (68.3%) and were from Oromo (67%) ethnic group. One hundred forty-six (46.8%) of the respondents completed grade 1–4. The median stay of street children on the street was 12 months with interquartile range (IQR) of 17 months. The median daily income of respondents was 35 ETB with interquartile range (IQR) of 20 Ethiopian Birr (ETB) (Table 1).

**Table 1:** Socio demographic characteristics of street children in Jimma town, Oromiya National Regional State, Southwest Ethiopia, March 2019

Variables	Categories	Frequency (***n*** = 312)	Percentage (%)
Sex	Male	281	90.1
	Female	31	9.9
Age	12–14 years	191	61.2
	15–18 years	121	38.8
Birthplace	Rural	225	72.1
	Urban	87	27.9
Religion	Muslim	213	68.3
	Orthodox	60	19.2
	Protestant	39	12.5
Ethnicity	Oromo	209	67
	Dawuro	31	9.9
	Amhara	39	12.5
	Keffa	33	10.6
Educational status	Never attend school	88	28.2
	Read and wright	23	7.4
	Grade 1–4	146	46.8
	Grade 5–8	55	17.6
Duration on the street (month)	1–12	182	58.3
	13–60	116	37.2
	> 60	14	4.5
Daily income (birr)	≤ 35	171	54.8
	> 35	141	45.2

### Family history of street children

Above 41 % (41.7%) of the respondents have lost either of their natural parent and 52.9 and 40% of respondents’ mothers and fathers can’t read and write respectively. Of the total children participated in this study, 44.5% of the children’s father work was farming. About 48.7 and 12.2% of respondents’ fathers and mothers were used substances respectively. Khat was predominantly used substance by respondents’ family. In addition to this 17.4 and 46.5% of respondents’ siblings and best friends use substances. Among users, 83.3 and 71.5% of siblings and friends use khat respectively.

### Substance use and reasons of engagement

According to this study 30.8% of the respondents use substances currently (Table 2).

**Table 2:** Distribution of substance use among street children, Jimma town, Oromiya National Regional State, Southwest Ethiopia, March 2019

Variable	Category	Frequency	Percentage
Substance us	Yes	96	30.8
	No	216	69.2

The study showed that 122 (39.1%) of the respondents used at least one substance in their lifetime. Among those, 77 (62.6%) used khat, 14 (11.5%) used alcohol, 57 (46.7%) used cigarette and 56 (45.9%) used mastics. Among current users, 63.5% of the respondents currently use khat, followed by cigarette, mastics, alcohol and benzene respectively (Table 3).

**Table 3:** Distributions of substances used by street children in Jimma town, Oromiya National Regional State, Southwest Ethiopia, March 2019

Substances	Ever used		Current use	
	Frequency	Percentage (%)	Frequency	Percentage (%)
Khat	77	62.6	61	63.5
Cigarette	57	46.7	42	43.8
Mastics	56	45.9	40	41.7
Alcohol	14	11.5	12	12.5
Benzene	8	6.6	4	4.2

**Fig. 1:**
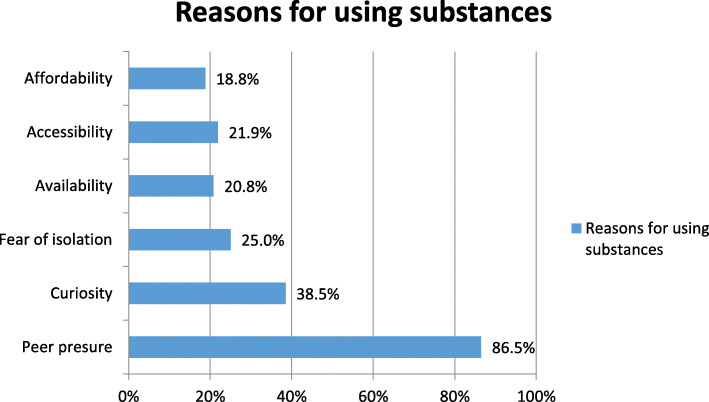
The bar graph showed that the reasons of using substance by the street children. The Y axis includes reasons of using substance while the X axis the percentage of respondents. Since the respondents can choose more than one answer, the total possibly more than 100%. The exact number is attached as a supplementary as “Supplementary 1”

Peer pressure (86.5%) and curiosity (38.5%) were reported as the leading reasons for substance use (Fig. 1).

### Bi-variable logistic regression of factors associated with substance use

Among all variables, age, sex, duration on the street, income, educational status, sleeping place (hotel veranda and old and abandoned buildings), child job (delivering message, carrying small items and begging) paternal substance use, maternal substance use, sibling substance use, best friend substance use had *p* value < 0.25 (Table 4).

**Table 4:** Bivariate logistic regression of factors associated with substance use among street children, Jimma town, Oromiya, South West Ethiopia March 2019

Variables	Categories	Substance use		COR(95% CI)	***P*** value
		yes	no		
Sex	Male	92	189	3.286 [1.117–9.668]	0.031*
	Female	4	27	1	
Age	12–14	49	142	1	
	≥14	47	74	1.84 [1.129–3.001]	0.014*
Educational status	Never attend school	34	54	1	
	Read and write	8	15	0.84 [0.325–2.211]	0.735
	Grade 1–4	38	108	0.56 [0.317–0.985]	0.044*
	Grade 5–8	16	39	0.652 [0.316–1.343]	0.246*
Daily income (birr)	≤ 35	47	124	1	
	> 35	49	92	1.40 [0.867–2.277]	0.167*
Sleeping place (Hotel veranda)	Yes	54	103	1.411 [0.870–2.288]	0.163*
	No	42	113	1	
Sleeping place (Old and abandoned buildings)	yes	22	82	0.486 [0.28–0.842]	0.010*
	no	74	134	1	
Child’s job (Carrying small items)	yes	75	150	1.571 [0.894–2.762]	0.116*
	no	21	66	1	
Child’s job (Delivering messages)	yes	8	46	0.336 [0.152–0.743]	0.047*
	no	88	170	1	
Child’s job (Begging)	yes	7	30	0.488 [0.206–1.153]	0.102*
	no	89	186	1	
Duration on the street	< 12	40	142	1	
	12–60	49	67	2.59 [1.561–4.318]	0.001*
	> 60	7	7	3.55 [1.176–10.716]	0.025*
Paternal substance use	Yes	52	100	3.64 [1.614–8.207]	0.002*
	No	8	56	1	
	Don’t know	36	60	4.2 [1.798–9.809]	0.001*
Maternal substance use	Yes	24	14	7.2 [3.383–15.322]	0.001*
	No	35	147	1	
	Don’t know	37	55	2.83 [1.620–4.929]	0.001*
Sibling substance use	Yes	28	26	3.06 [1.627–5.767]	0.001*
	No	45	128	1	
	Don’t know	23	61	1.07 [0.596–1.930]	0.815
Best friend substance use	Yes	76	69	8.096 [4.58–14.309]	0.000*
	No	20	147	1	

*COR* crude odds ratio; *CI* Confidence Interval; *p ≤ 0.25

### Factors associated with substance use

Age, educational status, maternal substance use, sibling substance use, best friend substance use and duration on the street were found to be significantly associated with substance use (*P*-value < 0.05) (Table 5).

**Table 5:** Logistic regression of factors associated with substance use among street children, Jimma town, Oromiya National Regional State, Southwest Ethiopia, March 2019

Variables	Category	Substance use		COR	AOR	95% CI AOR
		yes	no			
age	> 14	49	142	1.84	1.97	1.001–3.889*
	12–14	47	74	1	1	
Educational status	Never attend school	34	54	1	1	
	Read and write	8	15	0.84	0.86	0.242–3.08
	Grade 1–4	38	108	0.56	0.33	0.151–0.737*
	Grade 5–8 and above	16	39	0.65	0.27	0.093–0.756*
Maternal substance use	Yes	24	14	7.2	7.78	3.00–20.11*
	No	35	147	1	1	
	Don’t know	37	55	2.83	5.1	2.19–11.81*
Sibling substance use	yes	28	26	3.06	2.23	1.254–5.63*
	no	45	128	1	1	
	Don’t know	23	61	1.07	0.42	0.19–1.302
Best friend substance use	Yes	76	69	8.1	11.07	5.47–25.04*
	no	20	147	1	1	
Duration on the street (in month)	< 12	40	142	1	1	
	12–60	49	67	2.59	3.00	1.511–5.96*
	> 60	7	7	3.55	4.592	1.06–19.7*

*COR* Crude odds ratio; *AOR* Adjusted odds ratio; *CI* Confidence Interval *AOR doesn’t include null value

## Discussion

The study assessed the prevalence and factors associated with substance use among street children aged 12–18 years old in Jimma town, Oromiya, southwest Ethiopia. The overall prevalence of substance use in the last 1 month was 30.8%. This result was found to be higher than the prevalence among street children in Nepal [18] and Teheran, Iran [19]. The higher prevalence in the current study might be due to easily availability of substances in the area and lack of legal enforcement to prohibit the children from not consuming these substances. The result however was lower than that reported in Mekele [16], Assam district, India [17] and Western Nigeria [20]. The difference might be explained by a smaller sample size taken in the Mekele and the difference in cultural and socio-economic conditions for the other two studies.

This study revealed that, ever use of either of the substance was 39.1% and more specifically, 46.7 and 11.5% of the respondents had ever smoke cigarette and drank alcohol respectively. The result from the current study is different from that of a study conducted in Tehran, Iran [19] probably due to differences in settings, cultural norms, relevant policies and availability of the substances. Furthermore, within 30 days before conducted the study, the prevalence of khat chewing, cigarette smoking and drinking alcohol was 63.5, 43.8 and 12.5% respectively. Alcohol utilization was comparable with reports in Nepal [18] but the result is higher than reports from western Nigeria in that the prevalence of drinking alcohol and cigarette smoking 43.6 and 41.4% respectively [20]. This result is also lower than the study done in Brazil [21] and Nepal [18]. The possible explanation for this inconsistency might be attributed to different socio-economic status and difference in geographical locations of all the aforementioned studies.

This study showed that the odds of substance use was two times higher among respondents older than 14 years compared to respondents age 12–14 years. The result was consistent with studies done in Mekele [16] and Brazil [22] and similarities assessed by these three studies could be because of the likelihoods of the older children to exercise trialing with substances [23].

It is unquestionable that attending school makes a difference on abusing of substances by the street children and this fact was reassured by the findings of this study. Evidently speaking, those who attended grade 1–4 were by 67% less to use substances compared with respondents who never attended school. Similarly, respondents who were grade 5–8 were by 73% less to use substances compared to respondents who never attended school. The result was comparable with finding in Brazil [22]. The difference might be due to the fact that schools are one of the media for getting information about ill effects of substance use.

Another evidence uncovered by this study was that those whose sibling used substance were 2 times more likely to use substances compared to those whose sibling don’t use. The result is consistent with a result in Mekele city [16] and western Kenya [24]. The possible explanation might be siblings are thought to provide substantial reinforcement for anti-social activities like substance use.

Similarly, those whose best friend use substance were 11 times more likely to use substances compared to those whose best friend don’t use. This result was concordant with study done in Brazil [22] and western Kenya [24]. The probable reason might be older children use substances to avoid being stigmatized by their friends or to impress them or it might be due to similarity of the current methods applied with that of the studies conducted in the two countries.

Duration of stay on the street was among the factors identified as associated with substance use. Hence, those who stayed 1–5 year on the street were 3 times more likely to use substance compared to those who stayed less than 1 year on the street. The result is consistent with the findings reported in Brazil [22], Guwahati City, Assam [17] and western Kenya [24]. In addition, those who stayed greater than 5 year on the street were 5 times more likely to use substance compared to the counterparts who stayed less than 1 year on the street. These could be because of the inclination of the street children to use substances as a coping mechanism to survive on the street as they stay longer period than those who are staying shorter time.

Street children whose mothers use substance were 8 times more likely to use substances compared to those whose mothers were not using. Whereas, those who didn’t know about their mothers’ substance use status were 5 times more likely to use substances compared to those whose mothers do not use. This result is comparable with a study done in Mekele city [16], Kenya [24] and Guwahati City, Assam [17]. The possible reason might be street children are deeply influenced by people who raised up them.

### Limitation of the study

This study would have been quite complete, had it employed both quantitative and qualitative methods together but used only quantitative method and hence some important scenario about the problem were not explored deeply. On the other hand, because of ethical issue, the age group over which the study was done was limited to street children in the range of 12–18 years while there were many street children in the two who were younger than 12 years. Other limitation of the study was related to duration of time for which the subjects were asked about substance use status since the prevalence time period is short, there might be false positive; those street children don’t use substance regularly might be counted as user. On contrary, social desirability bias may underestimate the current prevalence of substance use.

## Conclusions

The prevalence of substance use among street children of Jimma town is high as compared to the figures in African and other countries. Age, educational status, maternal substance use, sibling substance use, using of substance by intimate friend and length of time on the street were found to be significantly associated with current substance use of the street children. Family has a crucial role in determining substance use behavior of the children. Governmental, non-governmental or charity organizations should work together to reduce the number of street children by responding to their needs and problems at least at three levels. The first level of intervention must be done at individual level and it requires targeting street children either as individuals or groups who are currently using substances, at risk of using them in the near future or at risk of sexual and reproductive health problems by building their basic skills, counselling and improving access to health. The second level of intervention is at community and family level and at this level, supporting community actions, providing services and ensuring the availability of resources for these children. The third general intervention can be termed as actions beyond the community and work places of street children. Educating and influencing these vulnerable groups through different mechanisms like advocacy of having safe and supportive environment at regional and national levels are also recommended.

## Supplementary information

**Additional file 1.** Ethical approval letter from Jimma University, Ethiopia.

**Additional file 2.** Ethiopian national research ethics guideline. We used the guideline as a bench mark to include street children aged between 12 and 18 years old.

## Data Availability

The datasets generated and analyzed during the study and we will make the data sets available to organizations and individuals based on reasonable request.

## Abbreviations

AIDS: Acquired immune deficiency syndrome
AOR: Adjusted Odds Ratio
CI: Confidence Interval
COR: Crude Odds Ratio
EDHS: Ethiopian Demographic and Health Survey
ETB: Ethiopian Birr
FSC: Forum on Street Children
IQR: Inter Quartile Range
JU: Jimma University
NGO: Non-governmental Organizations
SPSS: Statistical Package for Social Sciences
STDs: Sexually Transmitted Diseases
UN: United Nation
UNICEF: United Nations Children’s Fund
WHO: World Health Organization
