# Supplementary material for: Prevalence and factors associated with substance use among street children in Jimma town, Oromiya national regional state, Ethiopia: a community based cross-sectional study
Source: Subst Abuse Treat Prev Policy. 2020 Aug 20;15:61. doi: 10.1186/s13011-020-00304-3 (PMC7441729; doi:10.1186/s13011-020-00304-3)
Supplement: Supplementary file 1 — Additional file 1. Ethical approval letter from Jimma University, Ethiopia. [file 13011_2020_304_MOESM1_ESM.pdf]

JIMMA UNIVERSITY
ጅማ ዩኒቨርሲቲ

ቁጥር
Ref. No IHRPGD/750/2019
ቀን
Date 25/02/2019

Institutional Review Board (IRB)
Institute of Health
Jimma University
Tel: +251471120945
E-mail: zeleke.mekonnen@ju.edu.et

To: Mengistu Ayenew

Subject: Ethical approval of research protocol

The IRB of institute of health has reviewed your research project entitled:

"Prevalence and factors associated with substance use among street children in Jimma Town Oromiya, Ethiopia"

This is to notify that this research protocol as presented to the IRB meets the ethical and scientific standards outlined in national and international guidelines. Hence, we are pleased to inform you that your protocol is ethically cleared.

We strongly recommended that any significant deviation from the methodological details indicated in the approved protocol must be communicated to the IRB before they are implemented.

With regards!

Zeleke Mekonnen (PhD)
Associate Professor, Health Research and Postgraduate Director

Institute of Health
Jimma University

Generated by CamScanner from intsig.com
