# Supplementary material for: Prevalence and factors associated with substance use among street children in Jimma town, Oromiya national regional state, Ethiopia: a community based cross-sectional study
Source: Subst Abuse Treat Prev Policy. 2020 Aug 20;15:61. doi: 10.1186/s13011-020-00304-3 (PMC7441729; doi:10.1186/s13011-020-00304-3)
Supplement: Supplementary file 2 — Additional file 2. Ethiopian national research ethics guideline. We used the guideline as a bench mark to include street children aged between 12 and 18 years old. [file 13011_2020_304_MOESM2_ESM.pdf]

National Research Ethics Review Guideline

FDRE Ministry of Science and Technology

September 2014

Fifth Edition

National Research Ethics Review Guideline

FDRE Ministry of Science and Technology

September 2014

Fifth Edition

Forward

The success and sustainability of the country's economic growth depends, among others, on technology transfer supported by research and the subsequent benefit of research results.one of the critical issues enumerated in the Science and Technology Policy (STI) document is Science and technology research .The research activities and its result are expected to address social and economic problem of the nation.

The National Science and Technology Research Council (NSTRC), which was endorsed and established recently by the national Science, Technology and Innovation Council, will have immense contribution to address the national development plan. It will also contribute to align the research activities and its output with the need of the national development programme.

The expansions of higher education and research institutions have enormous contribution to the research activities undertaking in different sectors. One of the priority sectors which need particular attention and to be supported by research is the health sector. The purpose of most research undertakings in the health sector is to generate baseline data that will provide a strong basis for short and long-term action, while also considering the rights, safety, and wellbeing of research participants. I strongly believe that decisions on health issues matter such as, the investigation of diseases, treatment of individuals, and design of interventions for research participants need to be evidence-based. Fundamentally, research should be ethically sound and approved by the designated authorities in every country where the research may take place.

The National Research Ethics Review Committee (NRERC) is one of the committees established by the Ministry. Since the first national guideline for research ethics was issued in 1995, the NRERC has revised four times in order to keep up with the dynamic nature of research ethics.

I hope, this guideline will be applicable to all research activities conducted by all sectors and organizations in Ethiopia and thereby will have immense influence on building and strengthening an effective research system in Ethiopia.

Demiku Hambisa Bonsa
Minister

Acknowledgment

The Ministry of Science and Technology would like to thank higher Officials, Professionals and Donors for their contribution to enrich National Research Ethics Review guideline.

A special thank goes to the Centers for Disease Control and Prevention (CDC)- Ethiopia, the World Health Organization and the Ethiopian Public Health Association (EPHA) for technical and financial support provided during the revision of this Guideline.

We would also like to thank the consultants Dr. Solomon Kumbi, Dr. Yimtubeznashe W/Amanuel and Dr. Melesse Getu for their work in the revision of the fifth edition of NRERC Guideline.

Table of Contents

Forward **5**

Acknowledgment **6**

1. General **13**

1.1 Background 13

1.2 Introduction 15

1.3 Preamble 17

1.4 Scope of Application 19

2. Objectives **20**

2.1 General Objective 20

2.2 Specific Objectives 20

3. Ethical Principles **21**

3.1 Respect for Persons 21

3.2 Beneficence 22

3.3 Justice 22

4. Institutional Authority and Purpose **23**

4.1 Institutional authority 23

4.2 Purpose of NRERC, IRBs 23

4.3 Use of Policies, Procedures 24

5. Ethics Review System **24**

5.1 Organizational Structure 24

5.2 National Research Ethics Review Committee 25

5.3 Institutional Review Board, Level A 26

5.4 Institutional Review Board, Level B 27

5.5 Secretariat of the NRERC 28

5.6 Terms of Reference 29

5.7 NRERC Office 29

5.8 NRERC Operations 29

5.9 Registration and Accreditation 29

6. Establishment, Functions, Review Procedures **30**
6.1 Definition 30
6.2 Establishment 30
6.3 Composition 31
6.4 External Reviewers 32
6.5 Independence of IRB 32
6.6 Terms of Office 32
6.7 Roles and responsibilities of IRB Members 33
6.8 Orientation Education of Members 34
6.9 Termination of Membership 34
6.10 Dissolution of IRB 35
6.11 Committee Meetings 35
6.12 Mandates and Functions of IRBs 37
6.13 Informed Consent 37
6.14 Assent 38
6.15 Waiver of Informed Consent Documentation 38
6.16 Continuing Review 39
6.17 Suspension and Termination of Approval 39

7. Review Mechanisms **40**
7.1 General Requirement 40
7.2 Exempted Review Procedures 40
7.3 Expedited Review Procedures 42
7.4 Decision Making Procedures 44
7.5 IRB Decisions 44
7.6 Communicating Decisions to Applicants 45
7.7 IRB Records 45
7.8 IRB Review fee 46

8. Requirements for Submission to an IRB **46**
8.1 Administrative Requirements 46
8.2 Protocol Requirements 47
8.3 Types Of Research with Special Consideration 48

9. Human Biological Materials **57**
9.1 Definition 57
9.2 Acquisition, Storage, Secondary Use 57
9.3 Procedure for Exchange Transfer 58

10. Regulatory Oversight of Research **58**
10.1 Ministry of Science and Technolgy (MoST) 58
10.2 The Ethiopian Food Medicine and Health Care Administration and Control Authority 59
10.3 Institutional Review Boards (IRB) 59
10.4 Data and Safety monitoring Board 60
10.5 Institutional Bio-Safety Committee 62
10.6 Community Advisory Board 63

11. Monitoring Reporting **64**
11.1 Adverse Events: Definition, Grading, Follow-up, Reporting 64
11.2 Compliance Monitoring 66
11.3 Types of Monitoring Oversight visits 66

12. Research Misconduct **67**
12.1 Research Misconduct 67
12.2 Possible Actions Against Research Misconduct 68

13. Responsibilities of Investigators, Host Institutions, Sponsors **69**
13.1 Investigators 69
13.2 Host Institution 70
13.3 Sponsors 71
13.4 Donors 72

14. Networking **73**
14.1 Networking Mechanisms/Modalities 73

Bibliography	74
Appendix I. Acronyms	76
Appendix II. Glossary of Terms	77
Appendix III: NRERC Terms of Reference	85
Appendix IV: National Research Ethics Review Application Form	87
Appendix V. Research Ethics Review Form	88
Appendix VI. Material Transfer Agreement Form	89
Appendix VII. List of Participants to enrich NRERC Guideline	92

1. General

1.1 Background

Research involving human participants may have existed on Earth for several millennia, albeit the research may have been unscientific, uncoordinated, unmonitored, and largely unethical. Some of these experiments have survived the test of time and are still used wholly or in part, in modern healthcare and services. The development and safety of health-related services and practices evolved from experiments on self, other animals, and people in the community, although the ethics were far from acceptable in most cases.

On the other hand, the moral principles of ancient philosophers and practitioners are still used in modern day medicine, as well as in the field of ethics. One such example is the use of the Hippocratic Oath taken by physicians and health practitioners, though recently, the Oath has been challenged by contemporary philosophers and ethicists.

In Ethiopia, health research has been and is still being carried out by various sectors including government and private organizations, bilateral and multilateral agencies including United Nations (UN) agencies, and faith-based organizations. These organizations have been conducting research of their interest independently or through collaborations with institutions such as medical and paramedical schools, research institutes, drug control agencies, and others.

To disseminate these research findings, the grounds for formal peer-reviewed health research publication were laid by the Ethiopian Medical Journal in 1962. Other peer-reviewed scientific journals followed suit, namely, the Ethiopian Pharmaceutical Journal, the Ethiopian Journal of Health Development, the Ethiopian Journal of Health Sciences, and Ethiopian Journal of Reproductive Health.

Commitment of the government towards research and research ethics has been expressed in various undertakings throughout the years. These undertakings include:

- Endorsement of a constitution and legal codes that protect the well-being, welfare, and autonomy of its citizens, while also protecting the right to intellectual and academic freedom, as well as the pursuit of knowledge
- Establishment of medical and public health colleges since 1952, the Central Research Laboratory in 1942 which later was upgraded to an institute, the Institute of Pathobiology in 1966, the Armauer Hansen Research Institute in 1969, the Demographic Training and Research Center in 1982, and recently, the National Science Academy.
- Introduction of Ethics Review Committees (ERC) at the university level in the 1970s to protect the rights, safety, and welfare of research participants and to ensure independent review of protocols before commencement of research activities.
- Establishment of the Ethiopian Science and Technology Commission (ESTC) in 1975, with the mandate to guide, coordinate, and facilitate all Science and Technology-related activities including health-related activities. ESTC was later

changed to the Ethiopian Science and Technology Agency (ESTA) to lead the development and application of science, technology and innovation in Ethiopia, and further coordinate, regulate, and oversee research. On October 24, 2008, under proclamation No. 603/2008', ESTA was formally renamed as the Ministry of Science and Technology (MoST) by the Federal Democratic Republic of Ethiopia (FDRE).

- Development of a National Research Ethics Review Committee and the now dissolved, National Health Research Council, were formed to lay the foundation for standardized protection of human participants in research. National Health Research Ethics Review Guidelines were first developed in 1995 by the former ESTC. The guidelines were revised three times since its first release in 1995. At the institutional level, standard operative procedures (SOPs) for research ethics review came into practice by organizations that include, the Addis Ababa University Medical Faculty Institutional Review Board (AAUMFIRB), Armauer Hansen Research Institute (AHRI), Ethiopian Health and Nutrition Research Institute (EHNRI) now renamed Ethiopian Public Health Institute (EPHI), and the Ethiopian Public Health Association (EPHA).
- Launching a Health Science and Technology Policy in which the country's health research priority areas were explicitly listed as policy directives. The Health Science and Technology Policy in article 2.2.2.2 states the need *"to enhance the monitoring and coordination of Health Science and Technology activities and the practical implementation of research outputs*." This Health Science and Technology Policy in article 4.3.5 further stresses the need to *"Organize an ethical committee to review the ethical aspects and procedures of research undertakings as required"* as one strategy. In addition, article 4.3.6 emphasizes the need to "encourage institutional peer reviews....",
- Founding the Drug Administration and Control Authority in 1999 which was recently renamed the Food, Medicine and Health Care Administration and Control Authority (FMHACA)
- Declaration of the potential allocation of 1.5% of the gross national income or gross domestic product (GNI/GDP) to research in various disciplines.

To regulate and provide oversight for health research in light of its increasing landscape, complexity, and sophistication, MoST hired three consultants with expertise in the field of bioethics to review the existing guidelines. The experts reviewed local and international standards, guidelines, instruments, and procedures related to bioethics. Furthermore, opinions and suggestions were solicited from a dozen highly educated, ethically experienced, reputable professionals in the country. The shortcomings and strengths of the existing guidelines, extent of decentralization of protocol review, mandates of the National Research Ethics Review Committee (NRERC) and Institutional Review Boards (IRB), terminology, operative procedures, and general suggestions from these dozen professionals, was sought using a semi-structured tool. All efforts were made to strike a balance in accommodating the experts' sometimes differing opinions and suggestions for these guidelines.

Moreover, the draft guidelines were discussed with a wider group of experts and representatives drawn from the MoST, Ministry of Justice, Ministry of Health, FMHACA, IRBs of universities and research institutions, international and UN agencies, and other relevant stakeholders. The comments and recommendations from this nationwide consultative meeting were analyzed and incorporated before this guideline was endorsed and released for use to safeguard the rights and welfare of research participants and for oversight of research in Ethiopia.

1.2 Introduction

"Research is a systematic investigation including research development, testing, and evaluation designed to contribute to generalizable knowledge." Nevertheless, as a matter of principle, in research involving human participants, the well-being of the patient takes priority over the interests of society and the knowledge to be gained.

"Human subject [research participant] is a living individual about whom an investigator conducting research obtains data through intervention or interaction with the individual, or identifiable private information." Besides, research participants may include pregnant women where fetuses, fetal material, or abortuses are the primary subjects of interest. Similarly, research involving autopsy materials shall also be included as source of data.

The main purpose of health research involving human participants is for better understanding of the etiology, pathogenesis, and pathophysiology of disease and the related diagnostic, preventive, promotive, and therapeutic procedures and measures. Moreover, as evidence-based practice has become necessary at the turn of the 21st century, and quite understandably, existing procedures and practices are challenged continuously for their effectiveness, efficiency, quality, accessibility, and acceptability by the community.

However, in the quest to ensure better health and knowledge, the rights and welfare of research participants must be protected at all times. Participation must be voluntary and free of coercion, persuasion, manipulation, deception, undue influence or inducement, and threat or intimidation. The information shall be obtained in a private setting whenever necessary and confidentiality maintained throughout the lifetime of the research. This is even more important when research involves participants whose comprehension and decision making is compromised because of age, lack of knowledge on medical concepts and technological terms, or severe mental or behavioral disorders.

For all research involving human participants and human biological materials/specimens, investigators should be aware of, and are obligated to, respect and adhere to all ethical, legal, and regulatory requirements applicable in Ethiopia.

In this rapidly advancing, complex, and sophisticated era of genetic studies, preventive, diagnostic, and therapeutic clinical trials, as well as collaborative research and human biological material transfer being common place, oversight and regulation is imperative to properly safeguard the rights and welfare of human research subjects.

For research to be ethical, all of the following eight criteria must be met:

1. **Ethical justification and scientific validity:** The research must be rigorous in its methodology. For research to be ethical, the methods must be valid and practically feasible, the research must have a clear objective, be designed using sound scientific principles, have sufficient statistical power, and be based on adequate knowledge of the scientific literature.

2. **Science and social value:** The proposed protocol should demonstrate valid scientific basis/ground, enhance health or generalizable knowledge, and benefit individuals and the community where the research is conducted. However, the research participants' rights and welfare outweigh any benefit to the society or gain in knowledge.

3. **Favorable risk-benefit ratio to research participants and their communities:** Risks to subjects shall be minimized through using procedures that are consistent with acceptable research design and potential benefits enhanced. The maximum benefit should be provided at the lowest possible risk, and risks to research participants shall be reasonable in relation to anticipated benefits.

4. **Fair selection and enrollment of human subjects:** *"Scientific objectives, not vulnerability or privilege, and the potential for and distribution of risks and benefits, should determine communities selected as study sites and the inclusion criteria for individuals…"* The justification for selection and the equitable nature of selection of research subjects should be described.

5. **Privacy:** Privacy should be respected, confidentiality maintained, the opportunity to withdraw at anytime or refuse any component(s) of the research should be available, and the well-being of research participants should be monitored, while information related to research participants should be kept confidential.

6. **Independent/IRB review:** "*Individuals that are not affiliated with the research must review the research and approve, amend, or terminate the research.*" However, individuals involved in independent review with any conflicts of interest may be summoned to provide information to the IRB.

7. **Informed consent process:** The information provided to research participants should be complete and appropriate to the participants' level of understanding. The participant should be competent to give or refuse consent and research participants should provide their entirely voluntary informed consent without coercion, manipulation, undue influence, or intimidation.

8. **Community engagement:** Research is quite often generalized to the community from which individual participants are drawn from. Such generalization can result in different positive or negative impacts to the community in terms of stigma, resource drainage, health outcomes, and more. Hence, researchers are encouraged to involve the community in decision making about the design and conduct of the study. Besides, investigators should consider the local customs, traditions, culture and religious practices of the community where the research is proposed to be conducted.

1.3 Preamble

MoST attaches the highest priority to maintaining high standards of integrity, responsibility, and accountability in all research conducted in Ethiopia.

In addition, MoST obligates that *'Research with human subjects should be carried out only by or strictly supervised by, suitably qualified and experienced investigators and in accordance with a protocol that clearly states the aim of the research, the reasons for proposing that it involves human subjects, the nature and degree of any known risks to the subjects, the sources from which it is proposed to recruit subjects, and the means proposed for ensuring that subjects' consent will be adequately informed and voluntary. The protocol should be scientifically and ethically appraised by one or more suitably constituted review bodies, independent of the investigators.'*

MoST ensures that this guidelines basically operate within the legal framework of Ethiopia and that the IRBs operate independently and without influence and coercion. Furthermore, MoST demands that membership of IRBs should be large enough to ensure a robust discussion of protocols. Additionally, IRB membership should have a healthy mix of representation by different genders, disciplines, sectors, and laypersons.

While this document focuses primarily on guidelines for IRBs, it emphasizes that attention shall be given to the wider system of human research participants' protections of which IRBs are a part. These guidelines empower IRBs to perform effectively and efficiently with best intentions. The review guidelines are developed to operate in the existing research systems with the following objective realities:

- Constitutional and legal rights, relevant policies related to health and science research exist.
- These guidelines are executed under the Law of Ethiopia. IRBs operate under explicit legal authority.
- All research with human participants is subject to the oversight of an IRB.
- MoST has the primary responsibility for ensuring that IRBs are subject to adequate oversight.
- Mechanisms are in place to ensure IRBs work effectively and efficiently. IRBs are part of a larger research participant protection programs that also include training of IRB members and investigators.
- Procedures exist to ensure clear and efficient communication, harmonization of standards, networking, and cooperation among the NRERC and other IRBs, and between different levels of review and publication committees in departments/ units of institutions. In addition, procedures exist for the coordinated review of multi-site research within Ethiopia or international collaborative research.
- Mechanisms exist to ensure that IRB activities are coordinated with national regulatory authorities' oversight of drugs and medical devices.

- Mechanisms are created for obtaining community input into the ethics review system.
- A system exists for registration of IRBs that operate in Ethiopia.

MoST attaches emphasis that this guideline is part of an effort to establish, facilitate and strengthen an effective health research system in Ethiopia. The aim of the health research system is to advance and use of scientific knowledge to improve health and health equity. The key functions of this health system research are stewardship, of which research ethics is a part, finance of the systems, creation of and sustaining resources, and production and use of knowledge. Hence,

Understanding and considering

- The shortcomings of the existing guidelines in the face of the widening landscape of research areas, complexity of research procedures, and evolvement of genetic studies with the consequent biobanks,
- The critical importance of establishing a strong health research system,
- The relevance and responsibility of ensuring standardized research ethics review all over Ethiopia,
- The need to ensure independence of IRB operations and decision-making from influence by anyone who sponsors, conducts, or hosts the research it reviews,
- The need for ensuring networking and collaboration of IRBs, both local and international,
- The increasing number of health teaching institutions and with them the number and quality of research,
- Evidence-based practice is the order of the day, and

Cognizant of

- The rapidly advancing and complex research related to genetic, biomedical science, and transfer of human biological materials,
- The increasing number and scope of collaborative studies by numerous investigators and institutions within and outside of Ethiopia, and with the existing policy, academic and administrative environment, the increasing interest of donors to sponsor research in Ethiopia,
- Research participants' protection, that should involve not only research protocol review, but also ethically sound research participant-investigator interactions, continuous safety monitoring, adherence to approved protocol, and quality improvement in research,
- The duty to ensure mechanisms to make IRB operations transparent, accountable, consistent, and of high quality; establishing mechanisms for IRBs to employ reliable means to evaluate whether the staff and members routinely follow

the IRB policies, rules and guidelines; the need for both internal and external evaluation; the demand to address complaints and grievances from all parties involved in research (researchers, research participants, communities, sponsors, and others),

- That research should be carried out in full compliance with, and awareness of, standards, laws, regulations of the country, as well as local customs, community, and the mix of social, traditional, and cultural diversity,

MoST developed this National Research Ethics Review Guidelines to safeguard the rights, safety and welfare of all human participants in all research proposed to be conducted in Ethiopia, based upon the Health Science and Technology Policy and the responsibilities assigned to the MoST.

No other guidelines and requirements are allowed to diminish or remove any of the human research participants' protections set forth in this National Research Ethics Review Guidelines.

1.4 Scope of Application

This Guideline is applicable to all types of research that involves human participants, including but not limited to:

- Studies of a physiological, biomedical, biochemical, or pathological processes
- Genetic research, clinical trials, pharmaceutical, and other investigational products
- Research studies involving clinical records or other personal information
- Public health and epidemiological research
- Health systems research
- Quality improvement research
- Human health related behavioral research, research on medical and paramedical education, and research related to traditional healing.
- Research that may include one or a combination of observations; interviews, internet-based, mail-based, and telephone research; focus group research; survey and research with biological samples.

Furthermore, the guidelines are applicable in research proposed to be conducted by all sectors and organizations. These include, but are not limited to, public, private, faith-based, indigenous and international non-governmental organizations (NGOs), bilateral, multilateral, and United Nations' agencies.

2. Objectives

2.1 General Objective

The objective of this guideline is to safeguard the rights, values, and welfare of research participants through respecting the participants' autonomy, protecting the participants from harm related to research, and ensuring that individual/community benefits and fairness are maintained.

2.2 Specific Objectives

2.2.1. To ensure the rights and autonomy of research participants are respected and that participation or otherwise in research, is entirely voluntary

2.2.2. To safeguard research participants from unnecessary/unjustifiable risk

2.2.3. To ensure fair selection of research participants and fair distribution of benefits and risks

2.2.4. To create awareness among investigators, sponsors, reviewers, decision and policy makers, and individuals/communities on basic ethics principles

2.2.5. To ensure that research holds/embraces/possesses/considers social and cultural responsiveness/sensitivities for participating individuals and communities

2.2.6. To ensure the scientific integrity, ethical standards, and appropriate procedures for the conduct of research involving human participants

2.2.7. To monitor and evaluate ongoing and post-research ethical implications to the participants and the community, at large

2.2.8. To regulate human biological materials (sample/specimen) transfer

2.2.9. To ensure rigorous and robust ethical review of research protocols

2.2.10. To ensure timely review and communication of decision of submitted protocol to investigators

2.2.11. To facilitate cooperation and networking among IRBs at local and international levels

2.2.12. To comply with and regulate the complexity of research procedures

2.2.13. To put in place legal considerations against research investigators/ studies undertaken prior to appropriate ethics review or unethical implementation of studies.

3. Ethical Principles

High ethical standards in health research can be achieved only when investigators aspire to such standards in their research activities. To safeguard the rights and welfare of human subjects in research, all Research Ethics Review Committees (RERCs) and IRBs, regardless of their level, shall promote three basic ethical principles: 1) respect for persons 2) beneficence and 3) justice. In general, RERCs shall ensure that investigators have thought of ethical issues, specifically that no harm will be done and no resources wasted in the name of research, regardless of the research question planned for exploration. However, in certain circumstances, the weight given to each of these three basic ethical principles may differ in accordance with the type of the research and the setting where the research is conducted. Nevertheless, the IRB should ensure that the following basic ethics principles are met.

3.1 Respect for Persons

Out of respect, participants must be informed about the research and allowed to decide about participation. Research participants unable to make decisions independently deserve extra protection.

3.1.1. Autonomy - This principle aspires to protect the interests of human research participants from physical, psychological, and cultural harm. It refers to the obligation on the part of the investigator to respect each research participant as a person capable of making an informed decision regarding participation in the research study. It obligates investigators to respect the right of human subjects to hold their views, make choices and take actions without controlling influences. For human subjects who are not capable of making an informed decision because of age, mental, or medical capacity, the next of kin or guardian shall make the decision in the best interest of the research participant.

3.1.2. Informed Consent – The importance of informed consent of research participants is unquestionable and the informed consent should be analyzed in terms of containing the basic elements of information, comprehension, competence, disclosure, and voluntarism. Information shall be given in writing and signed or verbally approved by the research participant. The information to be provided should be written in a way that considers the local culture and values, as well as the level of understanding of the research participant. The information provided should weigh the research participants' competence. When informed consent is that of a third party (proxy; parent, next-of-kin, legally authorized representative (LAR), the reasons for the indirect approach shall be stated and become part of the protocol.

Research participants or persons giving proxy consent cannot give full informed consent unless the consent process/form contains adequate information. All such information shall be expressed in a language that is understandable to the participant. Generally, the consent form should explicitly indicate the points mentioned in **Section 6.13** below.

3.2 Beneficence

The principle of beneficence refers to the obligation on the part of the investigator to attempt to maximize benefits for the individual participant and/or community, while minimizing risk or harm to the individual/community. As much as possible, beneficence also considers inflicting no harm. An honest and thorough risk/benefit assessment must be performed. Balancing the risk and benefit of the research is indispensible in the design and conduct of the research.

Risk is the probability and magnitude of some future occurrence of harm. Harm is injury and setback to interests as a result of being a research participant. Risks to research participants include physical, psychological, and emotional harm. Risks can be known or presumed. And, although no specific regulations exist, risks may include physical, psychological, emotional, economic, educational, legal, and social harm.

In addition, beneficence includes whether the usual care is changed or manipulated to inflict no harm, minimize harm, remove harm, and maximize the benefit to research participants and to the community, or both.

3.3 Justice

Justice connotes fairness and equity in the distribution of the benefits and burdens of research to participants.

3.3.1. Justice demands equitable selection of participants, i.e., avoiding populations that may be unfairly coerced into participating, including but not limited to, prisoners, pregnant women, people with mental and physical disabilities, immigrants, refugees, ethnic minorities, marginalized groups and institutionalized persons, including children. There must be a justification for inclusion of these vulnerable groups in the research. There should be no disproportionate use of vulnerable populations. The same recruitment approach should be used in all populations. Injustice may arise when selecting participants from a specific socio-economic class, age, sex, racial, cultural, religious, creed, and institutional make up.

3.3.2. The principle of justice requires equality in the distribution of benefits and burdens among the population groups likely to benefit from the research. Distributive justice means that no segment of the population should be unfairly burdened with the harms of research. It thus imposes particular obligations toward individuals who are vulnerable and unable to protect their own interests. Conversely, distributive justice imposes duties neither to neglect nor discriminate against individuals and groups who may benefit from advances in research.

3.3.3. Justice also demands balancing the benefits and burdens to the community where the research is undertaken.

3.3.4. In addition, the investigators shall assure that information obtained in the course of the investigation remains confidential to protect partici-

pants from possible harm. Data unlinked from individuals or groups does not jeopardize confidentiality. The privacy of individual participants also needs to be protected throughout the investigation by the investigators.

4. Institutional Authority and Purpose

4.1 Institutional authority

NRERC and IRBs are established under the authority of the Ministry of Science and Technology, but function independently.

The MoST requires all research involving human participants or human biological materials to be reviewed and approved by NRERC or one of the MoST accredited and registered IRBs prior to initiation of any research-related activities, including recruitment and screening of participants.

4.2 Purpose of NRERC, IRBs

The NRERC and IRB's objective is to protect the rights and welfare of human participants in biomedical and behavioral research. The IRB reviews and oversees human participant research to ensure that it meets the ethical principles cited in this guideline, FMHACA regulations, and that it complies with legal requirements and other pertinent regulations, guidance, and local laws.

4.2.1. The NRERC and IRB's duty is to inform and assist the investigators and advisors on ethical and procedural standards related to the use of human participants in research, to facilitate compliance with this guidelines, Ethiopian law, and international regulations.

However, the primary responsibility for assuring that the rights and welfare of individuals are protected rests upon the investigators conducting the research. Others engaged in the conduct of the research including host institutions and sponsors share this responsibility. Faculty advisors serving as Principal Investigators (PIs) to students who conduct research have an obligation to carefully consider whether the students are qualified to safeguard adequately the rights and welfare of participants.

4.2.2. The NRERC and IRB have the authority to ensure that research studies conducted under its jurisdiction are designed and conducted in a manner that protects the rights, welfare and privacy of research participants. Specifically:

- The NRERC and IRB reviews, and has the authority to approve, require modification in, or disapprove all research activities that fall within its jurisdiction.
- The NRERC and IRB have the authority to conduct continuing review as it deems necessary to protect the rights, welfare, and privacy of research participants, including requiring progress reports from investigators.
- The NRERC and IRB may suspend or terminate approval of a study not being

conducted in accordance with the NRERC and IRB's requirements or that has been associated with unexpected serious harm to participants or others.

- The NRERC and IRB have the authority to observe or have a third party observe the informed consent process and/or audit the progress of any study in its jurisdiction as it deems necessary to protect the rights and welfare of human participants.
- The NRERC and IRB may place restrictions on a study.

4.3 Use of Policies, Procedures

The NRERC and IRB members and its Secretariat staff must maintain and follow all written policies and procedures consistent with Ethiopian regulations, good clinical practices, good manufacturing practices, and biomedical ethics when reviewing proposed research.

5. Ethics Review System

The MoST through the Ministry of Justice, shall ensure that health research ethics review is supported by an adequate legal framework that resonates with this guideline. MoST shall strive to put in place an appropriate and sustainable system to monitor the quality and effectiveness of research ethics review. In addition, MoST is responsible to ensure existence of mechanisms for networking and cooperation among IRBs at different levels, as well as IRBs of international standing in collaborative research.

All health research involving human participants must undergo review by the NRERC or an independent IRB. In Ethiopia, ERCs are established at three levels: National Ethics Commitee (NEC), Institutional (IRB Level A and IRB Level B). The composition and mandates of these ERCs are stated below. It is important to note that decentralization of the activities of the NRERC and subsequent establishment of new and empowerment of already existing Institutional IRBs, shall be realized.

5.1 Organizational Structure

To effectively and efficiently deliver research ethics review services, functional and structural arrangements are established at the National, and Institutional levels as depicted in the Organogram below.

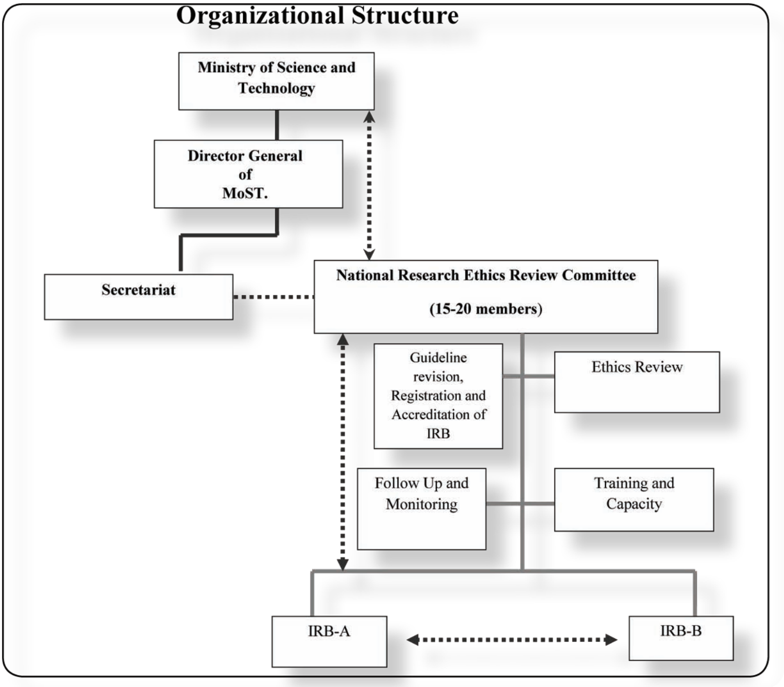

5.2 National Research Ethics Review Committee

The purpose of the NRERC is to safeguard the dignity, rights, safety, and welfare of all actual or potential research participants and/or communities. The NRERC is mandated to review research protocols and the supporting documents on their scientific and ethical merit. Furthermore, the NRERC is mandated to assure that proposed research resonates with the health research directives as stipulated on the Health Science and Technology Policy.

5.2.1 Composition

- Shall have members with professional competence and mix of varying backgrounds, gender representation, a community representative, research ethics training and experience.
- Minimum number of members is fifteen (15).

The details are described in **Section 6.3** below.

5.2.2 Mandates and Functions of NRERC

5.2.2.1. Develop research review guidelines and standards

5.2.2.2. Accredit and recommend licensing and registration of IRBs by MoST

5.2.2.3. Solicit funds to build capacity at all levels of IRBs

5.2.2.4. Organize and deliver research ethics training to IRBs at all levels using different media (classroom, on-site, internet-based); develop standardized training materials for countrywide use

5.2.2.5. NRERC Protocol Review - The NRERC is responsible for giving final ethical decisions on:

- All clinical trials involving new drugs, or new combinations of drugs, vaccines, new therapeutic regimens, and other biological products as well as invasive diagnostic procedures. For all research that involves investigational products, authorization and monitoring by FMHACA is a requirement before submitting a protocol for review and approval.
- Multi-center collaborative research, including student theses (MSc, PhD, specialization studies) that inherently exhibit more than minimal risk, and experimental/clinical trials.
- Research which is funded by companies producing the experimental product.
- Experimental research which is carried out by a national agency or agencies with international collaboration.
- Projects that require transfer of human biological materials (samples/specimens).

5.2.2.6. Umpire complaints, disputes, appeals and grievances on functions and review processes of IRBs submitted by researchers or institutions.

5.2.2.7. Monitor and evaluate IRBs at all levels.

5.2.2.8. Facilitate experience sharing among local and international IRBs.

5.2.2.9. Facilitate international registration of Level A IRBs such that their reviews are accepted by funding agencies.

5.2.2.10. Review of trials that are funded by manufacturers and pharmaceuticals,

5.2.2.11. Review very urgent research projects that are of national interest and priority, and

5.2.2.12. Policy-advocacy and creating community awareness on ethical principles in research, and legal and regulatory reforms, and changes related to research involving human participants.

5.3 Institutional Review Board, Level A

These IRBs can be regional or institutional. These are IRBs that have the capacity to review, monitor, document research protocols and undertakings involving humans in the region or the institution where they are based, or other institutions that do not have their own IRBs and beyond. Similarly, Level A IRBs shall be capable of safe-

guarding the rights, autonomy, safety, and welfare of human research participants.

5.3.1 Composition

- Shall have members with professional competence and a mix of varying backgrounds, gender representation, a community representative, research ethics training, and experience similar to or equivalent with the NRERC.
- Minimum number of members is five (5).

5.3.2 Mandates and Functions

5.3.2.1. Organize and deliver research ethics training to researchers in the region or the institutions.

5.3.2.2. Develop SOPs, that govern the IRB's research review procedures.

5.3.2.3. Submit progress report of the IRB's functions annually to the NRERC.

5.3.2.4. Inform the NRERC on occurrence of frequent, unexpected severe adverse effects (SAEs) related to research reviewed and approved by the IRB,

5.3.2.5. Review and approve research protocols similar to the NRERC except:

- Genetic research, stem cell research
- Research involving human biological material transfer outside of Ethiopia provided that the IRB notifies the NRERC Multicenter international collaborative research of experimental nature
- Investigation of new devices, drugs or vaccines not registered for use in Ethiopia
- Review of trials that are funded by manufacturers and pharmaceutical companies

5.3.2.6. Solicit funds to build its own capacity

5.4 Institutional Review Board, Level B

These can be regional or institutional IRBs. These are IRBs that have the capacity to review, monitor, and document research protocols and undertakings involving humans from the institution where they are based or other institutions that do not have their own IRBs, according to the mandates and functions stated below. Similarly, Level B IRBs shall be capable of safeguarding the rights, autonomy, safety, and welfare of human research participants. These IRBs may not have the expertise and experience of Level A IRBs, in which case the protocols can be reviewed by Level A IRBs of other institutions.

5.4.1 Composition

- Shall have members with professional competence and a mix of gender representation, a community representative, research ethics training, and experience,

which may not be similar to or equivalent with NRERC or Level A IRB.

- Minimum number of members is five (5).

5.4.2 Mandates and Functions

5.4.2.1. Organize and deliver research ethics training to researchers in the region or the institution.

5.4.2.2. Develop SOPs that govern the IRB's research review procedures.

5.4.2.3. Submit progress report of the IRB's functions annually to the NRERC.

5.4.2.4. Inform the NRERC on occurrence of frequent, unexpected SAEs related with research reviewed and approved by the IRB.

5.4.2.5. Review and approve research protocols that involve minimal risk.

5.4.2.6. Solicit funds to build its own capacity

5.5 Secretariat of the NRERC

The secretariat of NRERC reports to the Director General of MoST. The Director General of MoST is a voting member of the NRERC.

Responsibilities of the Secretariat:

- Receive applications from other IRBs.
- Ensure the completeness of application documents for ethical review.
- Distribute protocols to all ethics committee members and/or external reviewers, as applicable and when directed by NRERC chairman or delegate.
- Facilitate regular and extraordinary meetings in consultation with the Chairperson of the NRERC.
- Communicate decisions of the NRERC to the applying institution with a copy to the PI.
- Archive all project-related protocols, correspondence, decisions and minutes of the NRERC.
- Receive periodic progress reports from investigators and annual reports from other IRBs.
- Facilitate the accreditation and registration of IRBs based on the recommendation of the NRERC and endorsement by MoST.
- Propose revision criteria for registration and accreditation of IRBs.
- Support networking among the NRERC and other IRBs.
- Facilitate the monitoring and evaluation of the ethical implementation of research.
- Organize, support, and facilitate the conduct of research ethics training at National and Institutional levels.

- Manage and facilitate all official correspondence of the NRERC and
- Solicit funds for realization of the duties of the NRERC.

5.6 Terms of Reference

Each IRB shall develop its own terms of reference. The TOR of the NRERC can be found in **Appendix III.**

5.7 NRERC Office

5.7.1. Physical Location and Security - The NRERC should have a dedicated office located in the premises of MoST.

5.7.2. Only the NRERC Secretariat should be authorized to have access to the NRERC documents unless ordered by a court of law.

5.7.3. NRERC documents should be kept secure in locked cabinets and only accessed by authorized personnel.

5.8 NRERC Operations

5.8.1. The NRERC operations office should be spearheaded by the NRERC Secretariat as per instructions from the NRERC through the Chairperson or his/her designate.

5.8.2. The NRERC operations office should be separate and independent of the administration of MoST.

5.8.3. Applications to the NRERC must be channeled through the NRERC Secretariat.

5.8.4. All decisions and communication from the NRERC to the applicant must be conveyed through the Secretariat.

5.9 Registration and Accreditation

5.9.1. All IRBs in Ethiopia have to be accredited by the NRERC, and registered and licensed by the Secretariat of the NRERC at MoST.

5.9.2. Registration and renewal of all IRBs shall be done every two years from the date of registration or renewal of the IRB.

5.9.3. When IRB members are replaced for any reason, these outgoing members shall be notified in writing. The reason for termination of membership shall be clearly described in the letter.

5.9.4. Review procedures, TORs, and the review forms should be standardized.

5.9.5. The minimum acceptable number of members on any IRB is five. No IRB can have even numbered members for the sake of decisions, particularly when there is a need to vote.

5.9.6. IRBs shall have members with a varied professional mix and there

should be at least, one member from behavioral sciences, law, or humanities, be gender sensitive and ensure community representation by a lay person. No IRB can be composed entirely of a single profession, similar gender, or without a community representative.

5.9.7. Following registration, a letter of accreditation shall be given to IRBs by the Secretariat.

6. Establishment, Functions, Review Procedures

6.1 Definition

Institutional Review Boards (IRBs) are independent committees established in an institution to conduct initial and continuing review of research projects with the primary goal of protecting the rights and welfare of research participants. All institutions in Ethiopia that conduct research involving humans as research participants should set up IRBs in accordance with these guidelines. Where an institution cannot set up an IRB, that institution may rely on an IRB of another institution to review their research projects, provided the IRB is registered by the National IRB.

6.2 Establishment

6.2.1 Appointing Authority

- The Head of the Institution is the authority responsible for the appointment of IRB members. In cases where members come from diverse institutions, the appointment should be upon the recommendation of the institution where the potential IRB member is based.
- Members are selected in their personal capacities based on their scientific knowledge and expertise, as well as on their commitment and willingness to volunteer the necessary time and efforts for the Board's function.
- Appointments should consider age and gender distribution, and relevant but diverse professional representation.
- The appointing authority should write an appointment letter to the prospective member inviting him/her to be a member of the IRB.
- Members will sign a confidentiality agreement and conflict of interest (COI) form.

6.2.2 Applying to Establish an IRB

An institution that needs to establish an IRB shall apply in writing for approval and registration at the National Secretariat at MoST, and include the following requirements:-

- Statement that the IRB will follow the guidelines as stipulated in this document, law, relevant regulations.
- A list of IRB members identified by name, qualifications, profession, current

CV, representative capacity; any changes, in due process or in IRB membership must be reported to the secretariat.

- Written SOPs for the activities of the IRB: composition of IRB members; conducting initial and continuing review of research, and for reporting its suggestions/opinion and decisions to the investigator and the institution; expedited review process; follow-up and monitoring of approved studies.
- The NRERC Secretariat presents the application to the NRERC who shall examine the institution's application, and if satisfied, will recommend to MoST for endorsement of the registration and authorize in writing, the establishment of the IRB. The process of application and response from MoST should not exceed three months.

6.3 Composition

The IRB should consist of a reasonable number of members, who collectively have the qualifications and experience to review and evaluate the science, medical aspects, and ethics of the proposed research.

- Each IRB shall be composed of at least five (5) members, with varying backgrounds to ensure complete and robust review of research activities commonly conducted by the institution. It must be multidisciplinary and multi-sectoral in composition, including persons with relevant but diverse scientific expertise, balanced age and gender distribution, and who have the qualifications and experience to review and evaluate the scientific and medical ethics aspects of research protocols.
- All IRBs shall have at least one member whose main area of expertise is in the scientific field and at least one member whose primary area of expertise is in a non-scientific field.
- Each IRB shall include at least one member who is not otherwise affiliated with the institution or have a close family member working in the institution.
- The IRB should have at least one community representative, who does not necessarily have to have any scientific expertise, but may be a layperson that represents the interests and concerns of members of the community and is familiar with the community's values, customs, traditions, and culture.
- If an IRB regularly reviews research that involves vulnerable populations, such as children, prisoners, pregnant women, men/women in uniform or persons with disabilities, marginalized group, refuges, minorities, the IRB shall involve or coopt one or more individuals who are knowledgeable about and experienced in working with these research participants.
- All IRB members should at the minimum take one basic training on bioethics within one year of appointment.

6.4 External Reviewers

- If a protocol requires expertise that is beyond the competence of the IRB members or the IRB need additional opinion in the review process, the IRB may engage independent experts to review and give their opinion.
- The Secretariat should keep an updated list of experts along with their CVs, which should be reviewed annually by the IRB.
- Independent experts must sign privacy and confidentiality agreements and conflict of interest (COI) forms to ensure that the information in the protocol is protected and that consultants do not have any conflicts.
- The IRB may ask questions that could guide the review of the experts.
- The expert may be invited to attend or consult by telephone an IRB meeting but he/she cannot vote in the meeting.

6.5 Independence of IRB

6.5.1. The IRB must be independent from the appointing authority, hosting institution, investigators, sponsors and any other stakeholders in its review and decision-making processes.

6.5.2. If there is any conflict of interest, COI, regarding a particular protocol, IRB members must declare their COI and excuse themselves from the review process of that particular research protocol. This is critical to ensure an objective assessment of the protocol.

- COI can be declared at the time research protocols are submitted, upon receiving the IRB agenda prior to the meeting and at the beginning of each meeting.
- IRB members who have a COI related to any research protocol must not participate in any initial or continuing review of that specific protocol or related matters except to provide relevant, factual information which may be requested by the IRB. The conflicted IRB member cannot deliberate or vote on those protocols or related matters.
- The IRB member or invited expert with a COI will be required to excuse himself/herself from the meeting during discussion and decision.
- An IRB member or invited expert assigned to carry out an expedited review on a protocol (or related matter) for which a conflict has been identified must notify the IRB Chairperson or Secretary so that the protocol may be reassigned to another person.

6.6 Terms of Office

6.6.1. Membership should be for a period of two (2) years.

6.2.2. Membership may be renewed for two terms, however at least one-third

of the former members should be retained at every point in time. Thus, the maximum tenure of IRB members is four (4) years.

6.2.3. For continuity and the smooth operation of the IRB office, the IRB Secretariat may serve up to a maximum of five (5) terms, which translates to a maximum of fifteen (15) years.

6.2.4. For a permanently employed secretariat, tenure is not limited provided he/she is still under the employment of the IRB. If, in the event of additional qualified personnel being hired/trained, this time limit may need to be reviewed.

6.7 Duties and Responsibilities of IRB Members

6.7.1 Chairperson

- In order to enhance independence of the NRERC, the Chairperson should not be affiliated with MoST. In other IRBs, the Chairperson can be a member of the institution.
- The Chairperson should be selected by the IRB members through a process of nominations followed by secret ballot voting; the committee is obliged to inform the appointing authority.
- The Chairperson should have the authority to sign official IRB documents such as approval certificates.
- Should the Chairperson decide to step down as Chairperson of the IRB, he/she should inform the board in writing at least one month in advance; the committee is obliged to inform the appointing authority.

6.7.2 Vice Chairperson

- The Vice Chairperson should be selected using the same process as for the Chairperson.
- The Vice Chairperson should Chair meetings and sign official IRB documents when the Chairperson is not available.
- The Vice Chairperson may sign official IRB documents such as approval certificates if the Chairperson is not available.
- In the absence of both the Chairperson and Vice Chairperson, the IRB members should select an Acting Chairperson to chair the current meeting provided a quorum is satisfied.
- The selected Acting Chairperson should sign minutes of previous meetings confirmed during his/her Chairpersonship.
- The Acting Chairperson should not have authority to sign official IRB documents such as approval certificates but may sign minutes confirmed during his/her Chairpersonship.
- The process of resignation should be the same as that for the Chairperson.

6.7.3 Members

- Membership becomes effective upon accepting an invitation from the appointing authority. Acceptance must be indicated by the member's dated signature.
- A member should be willing to have his/her full name, profession and affiliation(s) published in the public domain.
- Members are responsible for reviewing protocols to safeguard the rights, dignity and welfare of study participants.
- Members are responsible for reviewing progress reports.
- Members are responsible for oversight visits in order to monitor ongoing studies approved by the IRB.
- Members are obliged to read protocols, including ancillary documents (e.g., patient brochures, informed consent forms, project reports and SAE reports given to them by the IRB Secretariat in advance preparation of IRB meetings.
- Members are obliged to keep IRB documents secure, private, and confidential.
- Members should attend IRB meetings regularly and participate fully and actively in deliberations.
- Members should participate in continuing education activities in research ethics, RE.
- Members must declare any COI for any protocol, and withdraw from the review of that protocol.
- Members must maintain privacy and confidentiality of documents and deliberations of IRB meetings.

6.8 Orientation Education of Members

- New members should undergo orientation training upon joining the IRB in order to familiarize them with basic human research ethics. Such training should be organized by the IRB Secretariat, the host institutions and/or any other players involved in such training.
- Continuous training of IRB members on health research ethics and other relevant areas such as Good Clinical Practice (GCP) and experimental designs should be conducted as necessary.
- Current CVs and records of training of all members should be kept on file by the IRB Secretariat in the IRB office.

6.9 Termination of Membership

- Membership may be terminated voluntarily. The member should write a resignation letter to the appointing authority through the IRB Chairperson giving at least a one-month notice.
- The Chairperson may resign by sending his/her resignation letter to the appointing

authority after informing the committee at its next meeting.

- Membership should be terminated by the appointing authority on the advice of the IRB if a member is going to be away for more than one year.
- Membership could be terminated by the appointing authority upon advice of the IRB if the member has been absent from five consecutive meetings without offering an explanation.
- Membership should be terminated by the appointing authority for misconduct that tarnishes the credibility of the IRB as determined by the IRB.
- Membership should be terminated if a member is convicted of a criminal offence.
- Membership should be terminated by the appointing authority in consultation with the IRB if a member is suffering from a chronic incapacitating illness that significantly reduces the ability to process information and make rational independent decisions.
- Membership should automatically terminate when a member dies.

6.10 Dissolution of IRB

The IRB should automatically cease to exist when the institution at which it is based ceases to exist.

6.11 Committee Meetings

6.11.1 Scheduled Full (Convened) IRB Meetings

6.11.1.1 The calendar dates and time of scheduled full (convened) IRB meetings should be agreed upon and confirmed by the committee and made public.

6.11.1.2 The frequency of the scheduled full (convened) IRB meetings should depend on the workload in terms of volume of applications submitted as well as the availability of IRB members who have other duties at their places of employment.

6.11.1.3 A quorum is more than 50% of the total number of IRB members.

6.11.1.4 The quorum should include members with the relevant expertise to effectively review the protocols on that days agenda. The presence of a non-scientist is required. The community representative, although not required, is essential.

6.11.1.5 The quorum should be present before a full (convened) IRB meeting is held.

6.11.1.6 The IRB Secretary should send an agenda; minutes of the previous meeting; notice about the date, venue and time of the next scheduled meeting; and other relevant documents to all IRB members at least ten working days before the meeting.

6.11.1.7 Applications should be submitted to the IRB Secretariat at least three (3) weeks prior to the next scheduled meeting that the applicant wants his/her applications reviewed.

6.11.1.8 The Secretariat should prepare and distribute a tentative agenda based on the applications received, matters arising from the previous meeting, SAEs, expedited reviews performed, continuing review applications, and amendments.

6.11.1.9 The general conduct of the meeting should be as follows:

- Meeting called to order by the Chairperson
- Adoption of the agenda, with or without changes
- Call for a vote to approve the minutes of the previous meeting
- Declaration of COI
- New business (new protocols)
- Other business
- Adjournment of the meeting by the Chairperson

6.11.2 Ad Hoc /Extraordinary IRB Meeting

6.11.2.1. Ad hoc/Extraordinary IRB meetings should be held if there is an urgent issue or issues that do not qualify for expedited review but require a full (convened) IRB meeting.

6.11.2.2. The Secretariat should circulate a notice giving the date, venue, time, and agenda of the ad hoc/extraordinary meeting at least 48 hours before the meeting.

6.11.2.3. The general conduct of the meeting should be as follows:

- Call meeting to order by the Chairperson
- Adoption of the agenda
- The issues for which the ad hoc meeting was convened

6.11.2.4. Relevant documents should be made available to IRB members at least 24 hours before the meeting.

6.11.2.5. A quorum must be present for the ad hoc meeting to conduct business.

6.11.2.6. Adjournment of the ad hoc meeting by the Chairperson

6.11.2.7. Minutes of the ad hoc meeting should be circulated at the next scheduled IRB meeting. A vote to approve or request modifications should be made.

6.12 Mandates and Functions of IRBs

The purpose of IRBs is to contribute to safeguarding the dignity, rights, safety, and well-being of all actual or potential research participants, by making an independent scientific and ethical review of research before commencement of research activities. Therefore, the major responsibility of IRBs is to safeguard the rights, safety, and well-being of research participants. The goals of research, while important, should never be permitted to override the health, well-being, and care of research participants.

IRBs should provide independent, competent, and timely review of the ethics of proposed studies. In their composition, procedures, and decision-making, IRBs need to have independence from political, institutional, professional, and market influences. They need similarly to demonstrate competence and efficiency in their work. They also need to ensure that there is regular evaluation of the ethics of ongoing studies that received a positive decision. Therefore, the functions of the IRB are:

6.12.1 Initial review

The IRB must determine that the following requirements are satisfied before it approves a research protocol:

- Risks to subjects are minimized. Risk to subjects are reasonable in relation to anticipated benefit (if any) and the knowledge that is expected to result
- Selection of subjects is equitable
- Determine that informed consent will be sought from each prospective study participant, or the participants' parent or guardian as appropriate
- Determine that informed consent will be appropriately documented
- Determine that there are adequate provisions to protect privacy of study participants and maintain confidentiality of all study related data
- Determine that there are additional safeguards included in the study to protect the safety and welfare of study subjects who are likely to be vulnerable to coercion or undue influence such as such as children, prisoners, mentally disabled people, etc.

6.13 Informed Consent

6.13.1. The IRB must determine that the consent is documented using a written or verbal informed consent form,

- The consent form should be approved by the IRB.
- The consent form must be signed by the participant, or a parent, or next-of-kin or a guardian and by the individual that conducted the informed consent process.
- In case the participant cannot read or write, a witness should sign that the consent process was carried out appropriately.

- A copy of the signed informed consent form should be given to the study participant.

6.13.2. Research participants or persons giving proxy consent cannot give full informed consent unless the consent process/form contains adequate information. All such information shall be expressed in a language that is understandable to the participant. Generally, the consent form should explicitly indicate the following:

- A statement that the study involves research, the purpose of the research, the expected duration of participants' involvement and identification of any experimental procedures.
- Foreseeable risks and discomforts.
- Reasonably expected benefits to the participants as well as the community.
- Disclosure of appropriate alternatives procedures or treatments.
- Extent to which confidentiality will be maintained.
- Compensation for possible injury if the research is greater than minimal risk; if so, an explanation is needed as to whether any medical treatments are available if injury occurs and what they consist of, or where further information may be obtained.
- An explanation of whom to contact for answers to pertinent questions about the research and research participants' rights, and whom to contact in the event of a research-related injury to the participant; A statement that participation is voluntary, that refusal to participate will involve no penalty or loss of benefits to which the participant is otherwise entitled. The consent form should also clearly indicate that research participants are free to withdraw from the study at any time and that the participant may discontinue participation at any time without penalty or loss of benefits. Research participants also are not obliged to respond to all questions in a study questionnaire.

6.14 Assent

Assent will be sought from a study participant under the age of 18 years old. The minor child should be given appropriate information based on the child's level of comprehension whatever the complexity of the research procedures. Then, an assent, a child's affirmative agreement to participate in research shall be sought from all children 12 years of age and above, in addition to the consent of a parent, next-of-kin, or guardian.

6.15 Waiver of Informed Consent Documentation

Waiver of informed consent or documentation of informed consent should be approved by the IRB. The investigator must secure an explicit waiver of consent from the IRB. The IRB may waive some or all of the elements of an informed consent and/

or a signed or thumb printed consent form for some or all of the research participants of a particular study, if the IRB determines that:

- The research project carries no more than minimal risk, If the research or demonstration project is to be conducted or approved by federal or regional government and is designed to study, evaluate, or otherwise explore public benefit or service programs; possible changes in or alternatives to those programs; possible changes in methods or levels of payment for benefits or services under those programs.
- The research project could not practically be carried out without the waiver or alteration (whenever appropriate the research participants will be provided with additional pertinent information after participation); In emergency/acute conditions where a new drug (or new combination of drugs) or a new procedure(s) potentially holds a direct benefit but, the research participant cannot give or refuse informed consent because of the acute condition, the IRB may waive need for informed consent. However, it is the responsibility of the investigator and the sponsor to publicize such a study to the community before commencement of any research activity. The IRB should monitor such publicity to ascertain the community's awareness.
- In emergency conditions of national or regional importance, for example epidemics, where a participant cannot refuse informed consent.
- In situations where deception needs to be applied to achieve the objectives of the study.
- The only record linking the research participant and the research project would be the consent document and the principal risk to the research participant would be potential harm resulting from a breach of confidentiality.

6.16 Continuing Review

- The IRB conducts continuing review of all approved studies at intervals appropriate to the degree of risk that the study participants are exposed, but not less than once a year.
- The IRB will require continuing progress reports annually , unless it designates otherwise.
- All changes in approved research projects should be reported and approved by the IRB before implementation, except where necessary to eliminate immediate apparent risks.

6.17 Suspension and Termination of Approval

The IRB has the authority to terminate or suspend its approval for research projects if it considers is appropriate such as:

- The research is not being conducted according to the approved protocol, or according to applicable guidelines,

- The research has been associated with serious harm to subjects, and
- The research creates a potential threat to the safety and welfare of research participants or the community.

The termination or suspension of approval should include a statement of the reasons verifying the IRBs decision and be reported to the investigator; the sponsor of the research and appropriate institutional officials.

Upon notification of the suspension or termination of the research, the investigator must promptly inform the research participants about the status of the research with assurance of continuing care and treatment.

7. Review Mechanisms

Each IRB must have written procedures, including procedures to be followed in their review mechanism. The following are the minimum requirements for an IRB review mechanism.

7.1 General Requirement

The IRB shall review proposed research at convened meetings at which more than 50% of the members of the IRB are present, including at least one member who represents the interests of the community.

- In order for the research project to be approved, it shall receive the approval of a simple majority of those members present at the meeting. The only exception to this procedure shall be in the case of expedited review as outlined in **Section 7.3.**
- Each IRB shall decide the frequency of its meetings, which should be announced.
- An IRB shall require that information given to research participants as part of informed consent complies with the general requirements for informed consent as prescribed by this guidelines.
- An IRB shall notify investigators in writing the outcome of the review of the research project. Such notice shall be provided to the investigator within 15 days from the date of IRB review of the research protocol. In case the IRB does not approve a research activity, it shall include in its written notification a detailed statement of the reasons for its decision.

7.2 Exempted Review Procedures

Research activities in which the only involvement of human participants will be in one or more of the specific categories stated below can be considered as exempt:

- Education: Research conducted in established or commonly accepted educational settings, involving normal educational practices.
- Education: Research involving the use of educational tests (cognitive, diagnostic, aptitude, achievement), surveys, interviews, or observation of public behavior, provided the research participants can not be identified.

- Research, involving the collection or study of existing data, documents, records, pathological specimens, or diagnostic specimens, if these sources are publicly available or recorded without identifiers. Or, if the information is recorded by the investigator in such a manner that research participants cannot be identified, directly or through identifiers linked to the subjects.
- Evaluation or examination of government projects or programs designed to explore public benefit or service programs; procedures for obtaining benefits or services under those projects or programs; possible changes in or alternatives to those programs or procedures.
- Taste and food quality evaluation and consumer acceptance studies where the foods are free of additives and where the ingredients are known to be safe.
- Emergency conditions of national or regional importance, such as epidemics.
- Quality assurance activities.

All research including that in the exempt categories must meet, at a minimum, the principles of respect for persons, beneficence and justice. The exemption determination other than mentioned above will be based on regulatory and institutional criteria and documented.

7.2.1. Exempt Study Submission Requirements - Research activities that meet the requirements for one or more exempt research categories must be endorsed by the IRB.

7.2.2. The investigator must complete the appropriate Exempt Application and submit the application along with (if appropriate):

- Questionnaires, surveys, assessments, interview questions, and tools.
- Consent statements, informed consent forms/scripts, and assent forms/scripts.
- Advertisements and letters of permission.

7.2.3. Exemption Categories and Determinations - Research activities in which the only involvement of human participants will be in one or more of the exempt categories, can be approved as exempt. The Chair or the Chair's designee, the IRB Secretary/Administrator, will complete the appropriate Exempt Category Form to review the protocol and make a determination.

7.2.4. Assessment of the research - The review of the research will also include:

- Whether the research has a sound research design
- Assuring there is minimal risk to participants
- Ensuring that the investigator has the resources, time, and expertise to conduct the study

The reviewer may require additional protections to meet the principles, including a level of informed consent appropriate to the research, or review by the full (convened) IRB.

7.2.5. Policies do not allow exemption of research involving video or digital recordings, and surveys or interviews that are extremely sensitive or personal. Allowance of audio recording is dependent on the research, is determined on a case-by-case basis, and must be documented.

7.2.6. Approval Period - At the one-year anniversary of the approval, an email is sent to the investigator requesting an update on the status of the study. During the approval period, the investigator needs to keep the IRB informed of any changes in the study, so that the IRB can ensure that the study continues to meet the exempt criteria. The investigator may close the study when data collection has ended or contact with the subject is complete.

7.2.7. Documentation of Exempt Review - If the study qualifies for exempt review, the reviewer will complete the appropriate Exempt Category Form which will be used as documentation.

7.2.8. Investigator and IRB members notification

- The investigator will be notified by appropriate medium of the exempt determination.
- Each month claims of exemptions will be listed on the IRB meeting agenda.

7.3 Expedited Review Procedures

Expedited review is review of a protocol that need not be seen by the full (convened) IRB, but by one or two IRB members assigned by the chair.

7.3.1 Eligibility Criteria

For the review to be expedited the proposed research must:

- Involve minimal risk, meaning that the probability and magnitude of harm or discomfort anticipated in the research are not greater in and of themselves than those ordinarily encountered in daily life or during the performance of routine physical or psychological examinations or tests.
- Appropriately protect privacy/confidentiality.
- Fall into one of the following categories:

1. Research employing survey, interview, oral history, focus group, or human factors evaluation.
2. Research involving materials (data, document, records, or specimens) that were originally colected for non-research purposes.
3. Collection of data from voice, video, digital or image recordings previously made for research purposes.

4. Research on individual or group characteristics or behavior (including, but not limited to, research on perception, cognition, motivation, identity, language, communication, cultural beliefs or practices, and social behavior).

- Follow-up on changes, amendments and annual renewals:
  1. Follow-up on changes or information requested by the Review Committee as a condition of approval.
  2. Minor amendments to previously approved research.
  3. Annual renewals, if the original approval was through expedited review.
  4. The only remaining activities involve long-term follow-up of previously enrolled participants.
  5. The only remaining activities involve data analysis.

- The review committee has determined and documented at a convened meeting that the research involves no greater than minimal risk (and there are no proposed changes that involve additional risk).

7.3.2 Applying for an Expedited Review

Requests for an expedited review can be submitted at any time to the Secretariat.

- An investigator who wishes to apply for an expedited review should submit an application indicating the reason(s) for the eligibility of the study for expedited review.
- Upon receiving an application for expedited review, the IRB Secretary/Administrator in consultation with the Chairperson/Vice Chair makes the initial assessment to determine if it qualifies for expedited review.
- If the study qualifies for expedited review, IRB chairperson or by one or more experienced reviewers designated by the chairperson from among the members of the IRB should be assigned to review the protocol. If the review involves a study amendment, the selected member should be a member who reviewed the previous version of the protocol.
- When the protocol is approved, the investigator(s) will be notified immediately through the Secretariat.

7.3.3. A summary of the protocols reviewed through the expedited process should be submitted to the full (convened) IRB at its next meeting.

7.3.4. A decision arising from an expedited review will be provisional pending approval from the Chair/Vice Chair or the IRB. Such decisions should be communicated to the investigator in writing.

7.3.5. Should a protocol be disapproved by the expedited review, it should be submitted for a full (convened) IRB review.

7.3. 6. The full (convened) IRB has the authority to confirm, modify or reverse a decision of the expedited reviewer. If the decision of the full (convened) IRB is contrary to the decision of the expedited review, detailed reasons and explanations should be recorded in the minutes.

7.3.7. The applicant should be informed of any modifications that the full (convened) IRB recommends and the ethical justification for such a decision.

7.3.3 Review Procedure

An expedited review covers the same issues as a full (convened) review. The reviewer has the same options as the full (convened) review committee, i.e., to approve, request modifications, or disapprove a protocol. An expediting reviewer, however, may not disapprove a protocol.

Expedited and exempt research: The chairperson should notify the IRB members about decisions made pertaining to such research using a suitable medium (full board meeting, internet, post, telephone). At that time, any member or the committee may request a re-review of the approved protocol at the full (convened) committee meeting. If this were to occur, the investigator would be notified and asked to suspend the study pending full review. Expedited reviewers refer the issue to the full committee if there is any question about the level of risk or the applicability of the activity categories before approving the protocol.

7.4 Decision Making Procedures

7.4.1. The IRB can only make decisions if a quorum is met.

7.4.2. A member with conflict must excuse himself/herself from the review process and voting.

7.4.3. Non-members such as project PIs and independent experts may be consulted as part of the review process, but do not vote.

7.4.4. Only IRB members who participated in the review process and deliberations should take part in the decision-making process.

7.4.5. IRB decisions shall be either unanimous or by consensus where there is a majority decision. If the decision is by voting the number for and against should be recorded in the minutes.

7.5 IRB Decisions

The IRB may reach the following decisions after reviewing the research protocol:

7.5.1. **Approved:** if a protocol fulfills all requirements as stipulated in this guideline or

7.5.2. **Approved on conditions:** if a protocol needs modifications.

7.5.3. **Not approved:** if a protocol is found to be unscientific and/or unethical.

7.6 Communicating Decisions to Applicants

7.6.1. Decisions regarding protocols should be officially communicated, in writing, to the applicant within 10 working days of the meeting where the decision was made.

7.6.2. Communication of the IRB decision shall include but not be limited to the following:

7.6.2.1. The name, title, and address of the applicant.

7.6.2.2. The exact title of the protocol.

7.6.2.3. The name of the study site(s) or study area.

7.6.2.4. The names and identification numbers (versions numbers/dates) of the reviewed documents.

7.6.2.5. A clear statement of the decision reached by the IRB.

7.6.2.6. The name of the IRB making the decision (letterhead of the IRB suffices),

7.6.2.7. The date of the decision and the signature of the Secretary/Administrator or Chairperson/Vice Chairperson.

7.6.2.8. In case of a conditional decision (approved on condition), any requirements by the IRB, including suggestions for revisions, should be clearly explained in writing to the applicant.

7.6.2.9. In case of a positive decision (approval), a statement of responsibilities of

the applicant and any requirements as stipulated in the decision by the ERC.

7.6.2.10. The validity period of the approval.

7.6.2.11. The final approval certificate/letter shall be countersigned by the Chairperson/Vice Chairperson.

7.7 IRB Records

7.7.1 Documentation

The IRB should maintain adequate documentation of all its activities, and maintain the following:

- File of detailed written procedures for the IRB.
- File of each protocol, containing all versions of protocol submitted and accompanying documents such as informed consent, approved versions, approval letters, progress reports submitted by investigators, reports of injuries to research participants, and other relevant documents.

- Agenda of IRB meetings, minutes of IRB meetings which shall be in sufficient detail to show attendance at the meetings; actions taken by the IRB; the vote on these actions including the number of members voting for, against, and abstaining; the basis for requiring changes in or disapproving research; and a written summary of the discussion of controversial issues and their resolution.
- Records of continuing review activities.
- Updated member files with CVs, training documents, etc.
- SAE reports submitted by investigators.
- Final reports from investigators.

7.7.2 Archiving

All closed files shall be retained for, at least, five (5) years after completion of the research project. All documents should be kept under a proper system that ensures confidentiality. All records shall be accessible for inspection by authorized personnel.

7.8 IRB Review fee

The Federal Ministry of Science and Technology will determine the condition and the amount of review fee for protocols.

8. Requirements for Submission to an IRB

There are requirements that must be fulfilled in order for the research protocol to be accepted by the IRB Secretariat for IRB review and approval. The requirements are administrative and research protocol related issues. Special categories of research entail fulfillment of particular requirements inherent in the type of research.

8.1 Administrative Requirements

8.1.1. The applicant must be the Principal Investigator (PI) or co-PI of the proposed research study.

8.1.2 A Protocol Application Form should be completed, signed, and dated by the PI/co-PI or his/her designee.

8.1.3. A signed cover letter from the PI or co-PI and the institutional details where the investigator is based (which should include a physical address, fax number, telephone number, mobile number and email address) also must be submitted.

8.1.4. The applicant should submit hard copies of the full research protocol and an electronic version.

8.1.5. All materials to be used in "advertising" the research study (e.g., campaign materials, brochures etc.) should be submitted along with the protocol.

8.1.6. Up-to-date signed and dated CVs of the PI and/or co-PI should be submitted. Also, bio-sketches of co-investigators should be submitted although full CVs may be requested by the IRB.

8.1.7. All applications should be submitted to the IRBs depending on the location of the research site.

8.1.8. The IRBs shall give final official approval for studies under their mandate. However, if an applicant has a complaint(s) against the decision of the IRBs, the study should be reviewed by the NRERC.

8.1.9. The NRERC or other appropriate IRB shall also review all studies under the NRERC's mandate and send the protocols with recommendations and remarks to the NRERC for final approval.

8.1.10. The Secretariat receives applications submitted using the application form from IRBs for ethics review and final decision as stated under NRERC mandates.

8.1.11. Upon receipt of complete applications, preliminary screening is done by the Secretariat.

8.2 Protocol Requirements

All research protocol and related documents submitted to the IRB for review and approval must at the least include the following information:

- Research protocol: title of the study, purpose of the study, sponsor of the study, background of the project, a rationale with full justification of the study and that there is no other alternative and less risky way of obtaining the data, description of the study population, participants inclusion /exclusion criteria, precise description of all proposed procedures and interventions, including the duration of the study, provisions for protecting privacy and confidentiality, provisions for managing adverse events, plans for data management including plan for statistical analysis and publication, and budget,
- Vulnerable population involvement requires further explanation to justify that without these vulnerable populations involvement there is no other way of obtaining the relevant data.
- All materials to be used (including advertisements) for the recruitment of research participants must be attached to the protocol.
- Data collection tools such as questionnaires, interviews /discussion guides, checklists and case report forms must also be submitted.
- Documents to be submitted for review include: Study protocol, protocol amendments, study/participant information sheet and informed consent form, current investigators' CV, investigator's brochure, and other materials to be used. The study informed consent process/form and information sheet, in both the offi-

cial language and, when necessary, its translation into the local language is required. A back translation into the official language may be requested by the IRB. In addition contact addresses of the PI, people to contact and the IRB that approved the research protocol must be included in the study information sheet.

- If the proposed study is a clinical trial, the investigator's brochure which provides an adequate summary of all safety, pharmacological, pharmaceutical and toxicological data available on the study product, together with a summary of the clinical experience of the study product (e.g. recent investigator's brochure, published data, summary of the product's characteristics etc.), must also be submitted. Information on payment and compensation available for research participants for time lost and injury or harm as a result of participating in the study.
- If the proposed study is a clinical trial, a certificate of Good Clinical Practice, of at least one of the investigators, and a certificate of Good Manufacturing Practice based on the nature of the research, must be attached.
- In studies that need transport of human biological substances, a request for a material transfer agreement, the type of biological material, how it is going to be processed and stored, how the material is intended to be analyzed, intent for possible future analysis, if there is one, as well as how and when it will be disposed of, must be included in the study protocol
- A dissemination and community involvement plan should be developed.

There are special categories of research that possess particular characteristics and demand special emphasis in ethical considerations. This type of research includes clinical/experimental trials, genetic, socio-behavioral, and collaborative research and research involving Vulnerable population.

8.3 Types Of Research with Special Consideration

8.3.1 Clinical Trials

8.3.1.1 Submission requirements

- An investigator's brochure which provides an adequate summary of all safety, pharmacological, pharmaceutical, and toxicological data available on the study product, together with a summary of the clinical experience of the study product (e.g. recent investigator's brochure, published data, summary of the product's characteristics, etc.), must also be submitted.
- If the trial involves an investigational product, prior approval and permission to import the investigational product must be obtained from FMHACA.
- Information on payment and compensation available for research participants for time lost and injury or harm as a result of participating in the research; Insurance certificate covering research-related injuries of participants and errors in protocol implementation,
- The PIs should attach a certificate of Good Clinical Practice (GCP), and a

certificate of Good Manufacturing Practice (GMP), based on the nature of the research. The investigator must assure that the clinical monitor and the IRB will have access to the participant's file to verify trial procedures and data are kept in accordance with the approved protocol. This should be stated in the participant information sheet. Additionally, the research participant should be told that by signing the informed consent, the participant is permitting access to the file by the clinical monitor and the IRB.

- SOPs for the trial; AE reporting format
- The investigator should affirm that he/she has sufficient time to conduct and monitor the trial, as well as to submit progress reports on time,

8.3.1.2 Investigator's File

The investigator must prepare a file containing documents related to the trial. During the study, the investigator is responsible for updating the file and regularly adding trial-related documents. Identification codes of subjects shall be kept for 10 years.

The Investigator's File should contain, at the minimum:

- Local regulatory requirements
- IRB and other authorities' written approval for all documents
- IRB and other authorities' written approval for protocol amendments
- Correspondence with the IRB
- Approved protocol and amendments
- All signed and dated informed consent forms
- Investigator's and Co-investigators' CVs
- Copy of the insurance certificate/other insurance
- Investigator's SOPs
- Notification and documentation of serious adverse events
- Specimen management procedures, trial supplies/equipment
- Participant identification list
- Investigator interim and final report/summary of the trial

8.3.1.3 Participant File

The participant file should, at the minimum, contain the following original information:

- Subject identification: family name, given name, date of birth, sex, and identification number in the trial
- Protocol identification number/study reference
- Dates of first screening and/or enrolment in the trial

- Name of drug or investigational product or procedure on test
- Dates of product administration and dosage, and procedure(s)
- Dates of assessment visit and name of individual responsible for making the assessment
- Serious adverse event and related treatment or medication
- Dates of laboratory specimen/sample collection.

8.3.1.4 Monitoring

- Compliance to approved protocol must be followed during the course of the research.
- Deviation from the protocol is acceptable only to manage/treat a serious adverse event that endangers the participant's life or is presumed to result into serious or permanent disability. In such acceptable cases of protocol deviation, the IRB, clinical monitor and the sponsor must be notified immediately. The protocol deviation must be appended to the approved protocol. And, the investigator should subsequently make a request for protocol amendment while applying for renewal of the research.
- The investigator should regularly submit a progress report; the progress report should include reports of the DSMB.

8.3.1.5 Medical Care for Trial Subjects

- The highest available standard of care shall be provided to trial participants
- In international collaborative research, the care should be equivalent to the care provided in the country where the sponsor or the collaborating institution is based.
- Adequate/standard medical care should be offered to participants for adverse events.

8.3.1.6 In case the trial is terminated or suspended prematurely, for any reason:

- The research participants must be informed with assurance of continuing care and treatment.
- The IRB and the sponsor should be informed as soon as possible.

8.3.1.7 In randomized, double-blinded trials, premature unblinding:

- The code may be broken because of serious adverse event, if deemed necessary by the DSMB during interim report,
- Must be documented in the file and reported to the clinical monitor immediately.

8.3.2 Genetic Research

Genetics Studies

Genetic screening should be distinguished from genetic testing at the outset. The terms are often used interchangeably, although they represent two different forms of genetic practice. Genetic screening is carried out on groups of people, which could consist of a section of the population defined by age, sex, or other risk factor, or a subgroup within the population, or within broad groups in which genetic factors may be responsible for certain disabilities.

Genetic Research: is defined as research conducted by investigators solely for the purpose of generating scientific knowledge about genes and/or the genetic basis of disease. Genetic data can be used for medical diagnosis, disease prevention, and population genetics studies.

Genetic Testing: A procedure to detect the presence or absence of, or change in, a particular gene or chromosome, including an indirect test for a gene product or other specific metabolite that is primarily indicative of a specific genetic change.

Genetic Screening: Large-scale systematic genetic testing offered in a program to a population or subsection thereof intended to detect genetic characteristics in asymptomatic people;

Genetic Counseling: A procedure to explain the possible implications of the findings of genetic testing or screening, its advantages and risks and where applicable to assist the individual in the long-term handling of the consequences; It takes place before and after genetic testing and screening:

- The protocol submitted should contain enough information on background, nature and scientific justification for the proposed study.
- In genetic studies involving acquisition of biological samples, the procedure to be used to obtain samples, the type and amount/size should be clearly stated. Issue of identifiers and how confidentiality will be maintained should be addressed.
- The collection, processing, handling, storage, transfer, and destruction of human biological materials and data should be conducted in a manner that protects the privacy of the participants and the confidentiality of their specimens and data.
- Procedures should be in place for the participant to request their sample be destroyed or stripped of identifiers. It should be stated that the study is for research purposes only and no individual results will be given back to study participants.
- The protection of the rights and welfare of the subjects should be a priority and confidentiality should be preserved to the fullest extent by the research team.
- The study participation information including research results will not be given to family members, employers or other third parties without written permission of the subject and IRB approval.

- Any shared samples must be anonymized to the recipient. Reimbursement of reasonable costs incurred by participants should not be of a magnitude so as to constitute an inducement to participate in the genetic study.
- During the informed consent process, there should be a clear statement that the study is for genetic research purposes. It is ethically imperative that clear, balanced, adequate and appropriate information shall be provided to the potential participant.
- The informed consent process should cover the human biological materials and data to be collected, data anticipated to be derived from the analysis of samples, and the health and other records to be accessed, their intended uses, storage, and duration of storage.
- The participants must be informed that specific information resulting from the study will not be available to participants and their families. Explanation should be given if the samples will be shared with third parties and that in these cases the samples will be anonymous.
- The participant should be given the option of allowing samples to be shared or not. All secondary and third party uses of biological samples should be restricted to **a**nonymized or anonymous samples. This means the sample cannot be identified or linked to an individual by the 'secondary-use-researcher' or the 'third party'. Limited, non-identifying, demographic information may be retained on the sample.
- Use of genetic materials beyond what is stated in the protocol must have the consent of the research participants or their representatives. In case of secondary use, i.e., research other than what it was collected, may be done only on anonymized samples and after getting prior approval by the IRB.
- Where subsequent use of human biological materials or data is envisaged that would not be consistent with the original informed consent, a new consent should be obtained from the participant/guardian/LAR or request for waiver of consent from IRB.
- The protocol should articulate clearly duration and place of storage of biological samples and data.

8.3.3 Socio-Behavioral Research

There are five potential risks in qualitative and behavioral research that the IRB, the investigator and the sponsor should be aware of:

- Anxiety and distress: qualitative research is aimed at in-depth understanding of sensitive topics with exploration of reasons and resilient issues. Involves open-ended questions and probing that give room for unexpected themes and variety of responses in the participant's own terms. Besides, responses are intimately dependent upon the context of the participants' beliefs, values, behaviors and actions. Hence, qualitative research possesses higher risk of anxiety and distress. Moreover, the differing experience of the participants largely makes

predicting who may experience the anxiety and distress difficult/problematic.

- Intrusion in life: exploring behavior related to health may require observation of participants at various places including the household, and sometimes, for a prolonged period of time.
- Exploitation: power imbalance between the researcher and the subject, or exerting undue influence.
- Misrepresentation: participants' opinion are taken out of context or interpreted in the researcher's perspective, dynamics of the qualitative interviews and the nature of data collected can be affected by the professional background of the researcher.
- Identification of participant: (by self or others) in published papers due to clues provided to describe the participant's age, gender, educational and economic status, place of residence, profession, etc.

Hence, to minimize these risks of harm, the following ethical issues should be given special emphasis during protocol review, conduct, and monitoring of qualitative research:

- Ensuring scientific soundness
- Organizing follow-up care where appropriate
- Considering obtaining consent as a process that should be subject to negotiation during and even after the end of the interview; the informed consent process should include auhorization of recording (audio, video) of interviews, focus group discussions, and observations.
- Ensuring confidentiality through securing storage of audio and video tapes and transcripts.
- Making 'respondent validation' of the researcher's analysis before dissemination of the research findings.
- Publicizing the research before commencement such that the community is aware of the observation that can be in public domains: at market places, sport events, brothels, theatres, etc. In such cases, the IRB may decide waiver of consent and documentation of consent.

8.3.4 Collaborative Research

Collaborative research is research that is conducted by investigators from more than one institution. If one of the collaborating institutions or investigators is based outside of Ethiopia, then, the research is termed "international collaborative research".

There are different potential reasons for conducting collaborative research. The reasons may include greater prevalence, convenience of or familiarity with the setting and the researchers, scientific or public health justification, and the research question or intervention is only relevant to the type of health problem in Ethiopia. Hence, the reason for choosing to conduct the research in Ethiopia should be explicitly stated.

8.3.4.1 Requirements

International collaborative research should be in line with the health research priorities of Ethiopia.

The research must be consistent with the Health Science and Technology Policy and must be responsive to the health needs of Ethiopians.

The research should contribute towards strengthening national research capacity to carry out similar research independently, including providing research ethics training to members of the investigation team.

The sponsor/donor should agree in advance that products will be made reasonably available after completion of the study, and the community must have access to the fruits of research.

All collaborative research must have a Principal Investigator or a Co-PI actively working in Ethiopia. Also, the PI or Co-PI must be employed or affiliated to a recognized institution or organization in Ethiopia. The research procedure should consider, to the extent possible, socio-cultural conditions in the community where the research is presumed to be conducted.

In all collaborative research, all involved institutions are required to review and write a recommendation of approval before submission to IRBs mandated to review and approve such collaborative research. In the event the involved institutions do not have a functioning IRB, the research protocol and other documents can be submitted directly to any of the IRBs mandated to review and approve collaborative research.

However, to avoid duplication of review efforts by IRBs, the NRERC may choose to conduct joint reviews in part or in whole, accept the review of another qualified IRB, or make other arrangements to establish oversight responsibilities.

Adoption of a paternalistic approach by research sponsors/donors towards institutions in Ethiopia or the research priorities of the government, is unacceptable.

8.3.4.2 Monitoring

When conducting collaborative research studies, each institution is responsible for safeguarding the rights and welfare of human participants and for complying with all applicable regulations. The IRB that reviewed and approved the collaborative research is primarily responsible for monitoring the ethical conduct of the research procedures. However, the National IRB can monitor the proper conduct of the research whenever it deems further oversight is necessary. Any modification, amendment, or change in the approved collaborative research protocol should be made at each collaborating institution. Material transfer agreements must be obtained whenever applicable.

8.3.5 Vulnerable Population

Vulnerable populations are those segments of the population whose capacity to safeguard their welfare, demand their rights and satisfy their interests, is compromised. Because of these limitations, they cannot provide or refuse consent. Vulnerable pop-

ulations are particularly subject to undue influence, manipulation, coercion, and intimidation. Hence, vulnerable populations deserve special protection from IRBs and other regulatory authorities.

8.3.5.1 Pregnant women and Fetuses

To conduct research on pregnant women, the following must be fulfilled:

- There is evidence from studies on pregnant animals and non-pregnant women that studies need to be done on risks related to pregnant women and fetuses.
- If the research holds the prospect of direct benefit to the pregnant woman and the fetus, and the knowledge cannot be obtained through other means.
- There should be no inducement to terminate a pregnancy.
- If the prospect of benefit is both to the pregnant woman and the fetus, an informed consent form from the mother alone suffices to enroll the woman into the research.
- If the prospect of benefit is solely to the fetus, informed consent should be obtained from both parents, provided they are competent to give or refuse the consent.
- In both conditions, the foreseeable risks of the research on the fetus or the neonate must be thoroughly explained.
- Viability of the fetus should be determined by a health care provider who is not a member of the investigation team.

8.3.5.2 Newborns/Neonates

In research involving neonates with uncertain viability, the following must be met:

- Previous research provides data to assess potential risks to neonates.
- Viability of the fetus should be determined by a health care provider who is not a member of the investigator team.
- There should be no other means of obtaining the knowledge to be derived from the research.
- There should be no added risk to the neonate due to the research.
- The research is likely to enhance viability and survival.
- Informed consent should be obtained from either of the parents.

For, research involving dead fetuses or organs or tissues of the dead fetus:

- There should be a prospect of important knowledge that can be possibly used in the prevention and treatment of similar or related conditions.
- Informed consent should be obtained from either of the parents of the dead fetus.

8.3.5.3 Children

Children are persons who have not attained the legal age for consent to treatments or procedures involved in research. The following apply in research involving children.

- The child should provide assent in addition to the informed consent by a parent or guardian as stated in **Section 6.14** above.
- The research presents a realistic opportunity to further the understanding, prevention, or alleviation of a serious problem affecting the health or welfare of children.
- Emancipated minors – working or earn their living, married, parenting – may be allowed to give an informed consent or an IRB may decide a waiver of consent.
- In research related to sensitive topics like drug use and abuse, sexuality, reproduction, STIs, where obtaining consent from a parent, next-of-kin, or guardian is challenging and may be problematic to the minor because of the nature of the research, assent with waiver of consent may be applicable.
- In institutional children, a legally approved guardian may give consent.

8.3.5.4 Prisoners

Prisoners may be under constraints because of their incarceration which could affect their ability to make a truly voluntary and decision which is not coerced, whether or not to participate as subjects in research. Therefore, additional safeguards should be included in the study to protect the rights and welfare of these subjects, as follows:

- If an IRB regularly reviews research that involves these vulnerable populations, consideration shall be given to the inclusion of one or more individuals who are knowledgeable about and experienced in working with these subjects.
- Where possible, a prisoner or an ex-prisoner co-opted in to the IRB reviewing the proposed research project should be included.
- Only research on conditions particularly affecting prisoners as a class and with reasonable probability of improving the health and well-being of the research participants should be permissible.
- The IRB shall determine that the informed consent process is properly applied with adequate assurance that a prisoner's participation or refusal to participate will not be considered in decisions regarding his or her release or further detention and each prisoner is clearly informed in advance that his or her participation in the research project will have no affect on his or her release.

8.3.5.5 Mental and Physical Disability

Persons with disability need special attention because they are prone to being socially marginalized. Therefore, their dignity, rights and well-being in research must be respected. Careful consideration should be made where proxy consent is to be used, and where the use of signed consent forms is not feasible, alternative viable methods should be employed.

Persons with disabilities (mental or physical) should not be unfairly excluded from participating in research. Researchers should make an effort to address communication, disability and comprehension constraints are often the excuse for exclusion.

People with mental disabilities or substance abuse related disorders include those people with psychiatric, cognitive, or developmental disorders. These groups of people are usually institutionalized and institutionalization may further compromise their ability to make voluntary decisions to participate in a research project. Therefore, research on people with cognitive disabilities or with substance abuse related disorders should:

- Provide sufficient justification for involving such people;
- Have appropriate evaluation procedures for ascertaining research participants' ability to give informed consent. If research participants are deemed unable to understand and to make an informed decision, then an appropriate proxy should be identified.
- Have an informed consent process that is free from coercion.
- Be of no more than minimal risk, or if minimal risk is involved, the risk is outweighed by the anticipated benefits of the research project to the research participant.

9. Human Biological Materials

9.1 Definition

Human biological materials include any substance obtained from a human research participant including, but not limited to, blood, urine, stool, saliva, hair, nail clippings, skin, and microorganisms, and other associated bio-products obtained from human research participants.

9.2 Acquisition, Storage, Secondary Use

The acquisition, storage, and future use of human biological samples from research participants in Ethiopia shall be guided by the following procedures:

- There should be a separate informed consent process for obtaining human biological samples for storage and for future use. This process includes the use of a consent form separate from that used for enrollment of research participants into the study.
- Research participants should know the purpose of sample storage, quantities of samples to be stored, place where samples will be stored, duration of storage, measures to protect confidentiality, policies that will govern use of the samples in future research, potential risks and benefits of storing samples for future research and any other information deemed necessary by the Investigators, IRBs.
- After explaining the need to store the samples, the research participant should be offered to choose whether their samples should or should not be stored for future studies.

- The host institution in Ethiopia should hold the samples in trust on behalf of the research participant.
- Research participants should reserve the right to withdraw their samples from storage if the samples are linked.
- Where samples have not been obtained as part of research (for example as part of routine surveillance, emergency procedures, laboratory quality control, notifiable diseases, routine counseling and testing, etc), the institution that collected the samples takes custodianship of the samples. Any future research study on such samples is subject to review by an IRB.

9.3 Procedure for Exchange Transfer

- When it is necessary to transfer samples for storage abroad, the host institution shall negotiate an appropriate contract with the recipient institution. This contract shall be in the form of a Materials Transfer Agreement (MTA). The specific details of the MTA should include, among other things, purpose for the transfer/export, clear arrangements for collaboration and benefit sharing, a framework for accessing and sharing data, restrictions to third party transfer, and annual reports to the host institution on the status of the samples.
- It is required that an Ethiopian scientist must be included as co-investigator in all future studies using the human biological materials collected from Ethiopia.
- The IRB in Ethiopia shall review all research studies on stored human biological samples.

10. Regulatory Oversight of Research

Regulatory oversight of research involving human participants is exercised at two levels. At the country level, the NRERC, which is established by MoST, is the primary responsible organ for oversight. At the institutional level, the organization's IRB, accredited by MoST, oversees all research being conducted under its jurisdiction. For research involving experimental/clinical trials, an additional approval to import and use the drug or investigational product should be solicited from FMHACA.

The legal obligations in research shall be overseen by MoST and FMHACA. IRBs, and DSMBs and are primarily responsible for safeguarding research participant safety. The Institutional Biosafety Committee (IBC) and Community Advisory Board (CAB) shall ensure public/community wellbeing.

10.1 Ministry of Science and Technolgy (MoST)

MoST is established by the 'Definition of Powers and Duties of the Executive Organs of the FDRE (Amendment) Proclamation No. 603/2008' on October 24, 2008. Under the Proclamation, MoST took over the rights and obligations of the ESTA and, under Article 20, is bestowed with the powers and duties to:

- Forward recommendations based on studies for adopting and revising policies, strategies, laws and directives on the development of science, technology and innovation activities.
- Prepare science, technology and innovation master plans; provide guidelines for…, programs and projects; monitor and evaluate their implementation.
- Set priorities for the country's research activities.
- Direct, coordinate and support science, technology and innovative activities, and countrywide research programs
- Support and strengthen institutions that undertake research and development activities.

10.2 The Ethiopian Food Medicine and Health Care Administration and Control Authority

The Ethiopian Food Medicine and Health Care Administration and Control Authority was established by 'Council of Ministers Regulation No. 189/2010' on August 23, 2010. Under the Proclamation FMHACA took over the rights and obligations of the Drug Administration and Control Authority (DACA) and, under Article 5, its '…objectives shall be to protect the health of consumers by ensuring: 1) food safety and quality 2) the safety, efficacy, quality, and proper use of medicines 3) competence and ethics of health professionals, medical practitioners and pharmacy professionals.

The '…authority is responsible to protect the safety and rights of the subjects participating in a trial and to ensure that trials are adequately designed to meet scientifically sound objectives.' 'The mandate of the Authority is to review protocols, and where necessary to protect the safety of subjects, to require protocol revisions and/or termination of trials. The Authority also has a right for on-site inspection of the quality of the data obtained…'

'Any clinical trial conducted in the country on human beings and/or on animals other than laboratory animals should have received prior permission before the commencement of the trial.' Furthermore, the Authority demands that findings from a clinical trial need its approval before publishing. Besides, the responsibilities of the investigator, the sponsor, and the monitor should be stated clearly in the research protocol.

The authority also requires that a specific application form for clinical trials shall be completed and submitted for review and approval of a research.

10.3 Institutional Review Boards (IRB)

IRBs are established by institutions whose mandate includes carrying out research. The primary function of IRBs is initial and continuing review, monitoring research to ensure adherence to the approved protocol in order to safeguard the rights and welfare of research participants, train faculty on research ethics, accredit, register, and monitor other IRBs, and develop guidelines applicable to all research in Ethiopia.

10.4 Data and Safety Monitoring Board

10.4.1 Definition

A Data and Safety Monitoring Board (DSMB) is an independent committee composed of a multidisciplinary group of experts established by the research sponsors to assess and report the ongoing scientific and ethical integrity of a study by reviewing and evaluating (unblinded as necessary) data at regular intervals.

- The DSMB should ensure that the study is conducted and the data are handled in accordance with the provisions of the research protocol and monitors adverse events and safety data.
- A DSMB should be established before the commencement of a clinical trial and its composition submitted to the IRB.
- All Phase I, Phase II, and Phase III, including drug efficacy and clinical trials proposed to be conducted in Ethiopia should have a safety monitoring plan, and a DSMB as recommended by an IRB.
- DSMBs should be established in studies where interim data analysis is required to ensure the safety of research participants. Other interventional studies, such as community trials, may be required to set up DSMBs on a case by case basis.
- Sponsors should consider the need for establishing a DSMB prior to undertaking a particular study. An ethics committee may also suggest to the sponsor that a DSMB be established for a particular study.
- Recommendations of a DSMB are communicated directly to the sponsor, but the sponsor should notify other relevant parties and ensure that the recommendations are communicated to, and acted upon by, the various parties involved in the research.

10.4.2 Composition

A Data Safety and Monitoring Board is composed of people external to the research team. The members of the Board:

- Should be independent of the sponsor and the manufacturer of the investigational drug or product.
- Have no conflict of interest in the research they are monitoring.
- Receive no scientific recognition in the form of publications or promotions from the results.
- Have relevant expertise (clinician with relevant specialization, clinical pharmacology and/or toxicology, epidemiology, statistics, ethics and additional types of expertise depending on the type of the research, e.g., anthropologists or community members for research which involve assessing cultural sensitivities).
- Have fair representation from participating countries in multi-center studies.

- Consist of at least three members and the size and necessary expertise of the DSMB will depend upon the research design.

10.4.3 Constituting a DSMB

When required by the nature of a study, a sponsor should ensure the establishment of DSMB to ensure the broadest possible coverage of potential research participants, and the validity and scientific integrity of the data. In order to generate competent reviews and sound recommendations, the DSMB should be multidisciplinary and include as appropriate, expertise in medicine (physicians with relevant backgrounds), clinical pharmacology and/or toxicology, epidemiology, statistics, clinical trial process, and ethics. The suitability of members of a board should be determined according to the nature of the study to be monitored.

The sponsor is responsible for establishing the DSMB's charter that defines the relationship between the sponsor and the DSMB, which should be included (or referred to) in the research protocol.

Members should not be affiliated with the sponsor, investigators, IRB reviewing the project, regulatory authorities, sites, or study staff. Members should also not have vested interest (e.g. a financial or other interest in an intervention or product similar to the intervention being studied).

A procedure should be established concerning the requirements for candidacy, including an outline of the duties and responsibilities of DSMB members

Procedures for reporting and addressing potential or real conflicts of interest for members and independent consultants should be clearly defined in the charter.

10.4.4 Responsibilities

- Review research protocol, informed consent documents and plans for data safety and monitoring.
- Evaluate the progress of the trial, including periodic assessments of data quality and timeliness, participant recruitment, accrual and retention, participant risk versus benefit, performance of the trial site, and other factors that can affect the study outcome.
- Consider factors external to the study when relevant information becomes available, such as scientific or therapeutic developments that may have an impact on the safety of the participants or the ethics of the trial.
- Review clinical center performance, make recommendations and assist in the resolution of problems reported by the PI.
- Protect the safety of the study participants.
- Conduct interim analysis of efficacy in accordance with stopping rules which are clearly defined in advance of data analysis.
- Ensure the confidentiality of the trial data and the results of monitoring.

- Assist the sponsor through remarking on any problems related with study conduct, enrollment, sample size, and/or data collection.
- Report on the safety and progress of the trial and make recommendations to the IRB and the sponsor regarding continuation, termination, or other modifications of the trial based on the observed benefits or adverse effects.
- Report the decisions to investigators who must submit those reports to the IRBs, which shall further report to the National IRB.

10.5 Institutional Bio-Safety Committee

10.5.1 Establishment

The IBC evaluates research projects that use recombinant DNA, agents that are infectious to humans, animals and plants, other potentially infectious materials, select agents and biological toxins. Institutional Bio-safety Committees (IBC) are established by institutions that undertake research on potentially hazardous substances of a physical, chemical, biological, or any other nature. Any institution involved in or planning to conduct research with potentially hazardous substances is required to set up or designate a competent IBC. Each IBC once formed shall consist of a bio-safety officer and at least three other officers with appropriate expertise in DNA, biological safety and physical containment. The IBC shall be certified by the MoST.

It is the responsibility of the Principal Investigator to notify and provide the IBC with the research proposal involving potentially hazardous substances of a physical, chemical, biological, or any other nature. The Principal Investigator is ultimately responsible for the registration, training, and safe handling of research materials handled by their personnel.

Members of the IBC shall protect confidentiality of all information given to them in the course of their work, and shall sign confidentiality agreements with their institutions.

10.5.2 Functions of an IBC

The IBC's function is to minimize potential human and environmental harm that may be associated with research on or with potentially hazardous substances such as pathogens, biological toxins, radioactive material and applications of bio-technology, especially recombinant DNA techniques and processes.

The purpose of an IBC is to ensure adequate containment of potentially hazardous biological agents, add a level of expert review and monitoring of potentially hazardous experiments, and provide a means of communication among researchers and health-care providers about potentially hazardous protocols.

IBCs shall:

- Notify NRERC and other IRBs of any research with potentially hazardous substances in their institutions.
- Conduct bio-safety review and approval of research proposals involving recom-

binant DNA and potentially hazardous substances.

- Continued review of approved research projects.
- Ensure the provision of suitable and safe storage and disposal facilities for all materials involved in work with potentially hazardous substances.
- Ensure that all appropriate technical personnel of the institution have adequate training in bio-safety.
- Establish a health-monitoring plan for all high-risk personnel involved in application, use and production of potentially hazardous substances.

10.6 Community Advisory Board

10.6.1 Establishment

Community Advisory Boards (CABs) are established by the investigator team. They are indispensable in orienting the investigator team about the local customs, traditions, terminologies, culture, and attitude towards research and development. Besides, CABs are important bridges to liaise the researchers with the community. CABs are critical forums to facilitate dialogue between community members, research participants and investigators.

CAB members shall be selected from the community where research is to be undertaken through a stakeholder consultative process. The CAB's role and expectations should be explicitly described in their terms of reference. Members of the CAB may include but are not limited to the following:

- Individuals familiar with local laws, customs, cultural values and gender issues
- Elders, opinion leaders, local chiefs
- Peer leaders, women leaders
- Religious leaders
- Representatives of the study population
- Media personnel
- Professionals who understand research or science issues

10.6.2 Functions

The main function of a CAB is to assist investigators with understanding and incorporating community concerns into their research procedures. The functions are expressed through different ways like advising on issues central to the informed consent process, achieving successful volunteer recruitment and retention, and other related issues.

The responsibilities of the CABs may vary according to the study location, size, complexity, familiarity of the investigators with the local setting, to mention but few. CABs functions are to:

- Provide information on traditional beliefs and needs of the study population and their concerns regarding the research project
- Provide input into the design of the research protocol as appropriate especially in the recruitment and the informed consent process
- Advise investigators on acceptable and effective methods for disseminating information about the research project and its outcomes
- Provide advice and support regarding retention of research participants including gender equity

11. Monitoring Reporting

Monitoring essentially encompass four activities: continuing review, review of the consent process, review for adherence to an approved protocol and review to identify unapproved activities. Continuing review is the most common, conventional, and fundamental aspect of monitoring of an IRB.

The primary function of monitoring is ensuring participant safety through assuring compliance with regulations and adherence to approved research protocols. In clinical trials, data monitoring is conducted to ensure data quality and safety. The DSMB is designated by the sponsor upon the recommendation of the IRB primarily to periodically monitor data quality and participant safety. Moreover, interim analysis of risks and benefits should be a component of monitoring to ensure safety, whenever applicable.

In multi-center research, monitoring at each of the research centers should primarily be carried out by the local IRBs. However, the NRERC is also responsible for oversight at selected, representative research centers, as deemed necessary.

In international collaborative research, periodic progress report must be submitted, and all adverse events must be notified to the NEC.

The investigator bears the major responsibility of regular monitoring and reporting of adverse events to the IRB. The sponsor is also obligated to report, or otherwise, ascertain the adverse events are reported to the IRB.

Based on monitoring and reporting that includes Adverse Events (AEs) as applicable, the IRB may allow continuation, suspension, or termination of the research or recommend an amendment (a change) in the research procedure. Besides, if the research involves a drug or investigational product, the investigator must notify FMHACA.

11.1 Adverse Events: Definition, Grading, Follow-up, Reporting

11.1.1 Definition Grading of an Adverse Event

Adverse events are considered to be serious when they are fatal, life threatening, cause serious or permanent disability, cause or protract hospitalization, cause congenital anomaly, or lead to death.

An AE is considered to be related with the research procedure, drug and/or an investi-

gational product based on temporal association with and response pattern to the procedure, drug or product, and the relationship, or otherwise, to the research participant's clinical state, other interventions unrelated to the research or concomitant therapy.

An AE shall be considered unexpected if the event has not been observed or documented in a similar research involving humans; the characteristics or severity of the event is inconsistent with information in the investigators brochure; or the event is observed with higher frequency or severity than previously documented.

An adverse event is graded as:

Mild: if the event does not interfere with day-to-day activities and does not require treatment.

Moderate: if the event marginally affect the day-to-day activities but can be tolerated by the participant or require out-patient treatment.

Severe: if the event significantly interferes with daily activities and demands hospitalization or procedures for relief.

11.1.2 Follow-Up on an Adverse Event

Once an AE has occurred, the investigator shall:

- Monitor the AE closely
- Provide a standard care to manage the AE and follow the AE until complete resolution of the AE.
- Thoroughly investigate the likelihood and extent of relationship of the AE with the research procedure, drug and/or investigational product.
- The details of the AE, from occurrence to complete resolution, must be recorded and attached to the participant's file.

11.1.3 AE Reporting

- A serious adverse event and the measures taken to manage the AE must be reported by the investigator, in writing, to the IRB Secretariat and to the clinical monitor, if one is assigned by the sponsor, within 48 hours of occurrence of the event, even if the AE is considered not to be related to the research procedures. Mild adverse events should be reported within five working days of occurrence.
- The IRB examines the AE report and the appropriateness, or otherwise, of the measures taken, and whether the measures are in accordance with the approved protocol. Measures beyond the approved protocol shall be documented in the participant's file and reported to the IRB. The IRB ultimately decides the need for additional action.
- The medium for reporting should be according to the communication means/channel described in the approved protocol.
- Anonymity of the research participant shall be respected when all information are sent/reported.

11.2 Compliance Monitoring

Progress reports - PIs should submit progress reports at regular intervals stipulated by the IRB as a condition for renewal of ongoing research. Periodic progress reports enable the IRB to determine whether the research is progressing according to the approved protocol. In clinical trials, the progress report should include reports of the DSMB.

The IRB shall establish a follow-up mechanism to monitor the progress of all ethically approved research. The follow-up review shall be done in the following manner:

- Ethics approval is valid for one year. For research that takes more than a year to complete, a renewal (continuation) application should be submitted to the Secretariat with a full progress report and justification for IRB approval.
- Any amendment in the protocol at any time should be reported to the Secretariat and approval secured from the IRB.
- Serious and unexpected AEs shall be reported to the Secretariat as stated above in **11.1.3**
- In case of a premature suspension or termination of a research, the investigator should notify the Secretariat including the reasons for the premature suspension or termination of the research and summary of the research findings.

11.3 Types of Monitoring Oversight Visits

11.3.1 Types of Monitoring

Passive monitoring – The IRB receives information about the research that it approved and uses that information to assess the study's progress.

Active monitoring – IRB members should physically visit the research site(s) in order to assess the conduct of the studies.

- Approved research should be actively monitored to ensure adherence to ethics principles, as considered necessary by the IRB.
- IRB members should use the IRB's oversight checklist in order to ensure appropriate issues are assessed during the visit.
- Should research exist that has not received ethical clearance, the presence of such research should be proved through a site-visit, witnesses, and appropriate and prompt actions should be taken (see **Section 12.2**).
- The number of IRB members needed to conduct an oversight visit depends on the workload of the monitoring team. To maximize objectivity, at least two (2) members of the ERC or delegated persons with diverse expertise and drawn from different institutions should make up the monitoring team. A monitoring team may include the community representative in the IRB.

11.3.2 Types of Oversight Visits

The type and frequency of oversight visits should depend on the level of risk and complexity of the research. The IRB can make the monitoring visit announced or unannounced.

IRB –initiated announced oversight visit: the IRB informs the PI the date of the visit in advance.

IRB-initiated unannounced oversight visit: the IRB does not inform the PI in advance of the visit.

Additional monitoring visits may be made for the following reasons:

- Response to reports made directly to the IRB or circulating in the community.
- Increased frequency of SAE reports.
- Failure to submit progress reports or a final report in time.
- Reports of suspected research misconduct.
- Investigators who extend their research beyond the approved time frame without formal approval from the IRB.
- Investigators that are suspected of having changed their objectives and study design without the IRB's approval.
- Any other reason that the IRB feels warrants further follow-up.

12. Research Misconduct

All researchers are obliged to respect the requirements set in this guidelines and the law and regulations related to research. Misconduct in health research is one of the aspects that make research unethical.

12.1 Research Misconduct

Research Misconduct includes but is not limited to:

- Conducting health research involving human participants or that which potentially affects humans without first obtaining ethical approval.
- Collecting samples or information from human participants without first obtaining valid, voluntary informed consent except in conditions where waiver of informed consent is applicable.
- Sharing with other investigators samples collected from human participants or institutions without ethical approval and without a signed Material Transfer Agreement.
- Sharing samples collected prospectively from human participants with other investigators or institutions without the informed consent of the participant. The IRB may waive the requirement for informed consent in the case of archived

and anonymized samples if the justifications are considered to be ethically and scientifically sound by the IRB.

- Failure to submit mandatory reports such as SAE reports, progress reports and final reports to the IRB.
- Failure to uphold the confidentiality of research participants' information including informed consent documents.
- Failure to report deviations from the approved protocol procedure(s) in time. Deviation from approved protocol procedure(s) should not be made without the agreement of the IRB that approved the protocol except when it is necessary to avoid immediate danger to a research participant.
- Unjustifiable deviations.
- Fabricating or falsifying data, or knowingly plagiarizing others' work.
- Misuse of research funds.
- Forgery of IRB documents (e.g., alteration of approval letter/certificate; Material Transfer Agreement, etc).

12.2 Possible Actions Against Research Misconduct

The IRB may receive reports of cases of misconduct via investigators, community members, voluntary whistle-blowers, research participants, or through its oversight activities. Upon receiving such reports, the IRB should confirm the validity of the alleged misconduct before deciding on an appropriate course of action. The actions that the IRB may take after confirming the misconduct include:

12.2.1. A letter of warning written to the PI by the IRB Chair with instructions for the misconduct to be stopped and/or rectified. The head of the institution, partners, and sponsors should be copied.

12.2.2. Corrective or educational measures.

12.2.3. Frequent monitoring of research activities.

12.2.4. Recommended and more frequent reporting by the investigator of his/her research activities.

12.2.5. Suspension of eligibility to receive research grants. The IRB may blacklist the investigator for a period of time to be determined by the IRB. During that period the IRB should not approve any research protocol submitted by the blacklisted investigators. The list should be copied to the relevant authorities.

12.2.6. In the event that serious harm/injury was caused to participants as a result of the misconduct, compensation for the harm/injury should be made by the investigators, or the host institution, or both. All research-related harm or injury as a result of ethically unapproved research shall be compensated by the investigator, the host institution,

or both. The compensation package should be determined by qualified and relevant authorities.

12.2.7. Temporary or permanent suspension of the PI and other investigators from research and/or professional practice.

12.2.8. Suspension of all research being conducted by the investigator.

12.2.9. Termination of the research.

12.2.10. The host institution may also be temporarily suspended from research activities.

12.2.11. Editors of journals should refuse publication of manuscripts from unethically conducted research and retract articles that are already published but eventually found to be conducted unethically.

12.2.12. In the case of criminal misconduct inform legal authorities.

13. Responsibilities of Investigators, Host Institutions, Sponsors

Responsible conduct of research requires that all stakeholders discharge the duties expected from them according to this guidelines and the law and regulations of Ethiopia.

13.1 Investigators

The investigator is responsible for overall conduct of the research according to the approved research protocol/procedures. More specifically, the investigator:

13.1.1. Shall maintain adherence to basic ethics principles,

13.1.2. Shall possess appropriate scientific and human ethics standards,

13.1.3. Shall ensure the highest possible standard of health care and follow-up, within the limit of the investigator's and the sponsor's capacity, is available to research participants; the available care shall be provided for a variable period even after the completion of the research based on the researched disease, condition or instrument; shall open an investigator's file where all documents related to the research are kept.

13.0.4. Shall keep records of informed consent document confidentially (in a locked cabinet).

13.1.5. Shall ensure privacy and confidentiality is maintained for research participants. The investigator shall ensure that hard data is kept in locked cabinets and electronic data is password protected accessible only to appropriate personnel.

13.1.6. Monitor research staff to ensure the research is done according to the approved research protocol/procedures.

13.1.7. Periodically submit a progress report to the IRB. The frequency of the report is to be determined according to the level of risk inherent in the

research, i.e., the higher the risk the shorter the reporting interval.

13.1.8. Shall promptly investigate serious adverse events and take appropriate measures to safeguard the safety of human subjects. The investigator shall inform such adverse events and measures taken, if any, to the IRB, clinical monitor and the sponsor.

13.1.9. Shall inform the IRB and obtain approval for any changes or amendments in the approved protocol/procedures except in circumstances where an apparent immediate hazard or danger to the research participants. Any amendment shall be appended to the approved research protocol.

13.1.10. Shall inform the IRB, clinical monitor, the sponsor and the participants if the study is terminated or suspended at any time during the research process.

13.1.11. Shall be responsible for periodic assessment of the quality of data management as well as reporting on interim analysis whenever appropriate.

13.1.12. In case of clinical/experimental trials, shall ensure, at least, one of the investigators have a certificate on good clinical practice and/or good manufacturing practice or both or whichever is appropriate.

13.1.13. Shall ensure beneficial investigational products are available to the community after the research is completed.

13.1.14. Shall report to the DSMB, as applicable.

13.1.15. In collaborative research, shall consider the cultures and ethnic diversities and should make the research objectives particularly clear and remain aware of the concerns and welfare of the individuals or communities to be studied.

13.1.16. Shall provide adequate information in all publications to the reader, and to colleagues to permit the methods and findings to be properly assessed. Limits of reliability and applicability should be made clear.

13.1.17. Shall submit final report and findings to the IRB.

13.1.18. Shall ensure the community where the research is conducted is informed about the research findings.

13.2 Host Institution

The institution's culture in which research is conducted strongly influence whether ethical conduct of research is supported or valued. The host institution must work closely with the investigator. The host institution shall monitor the investigator(s)' research activities at the institution. More specifically, the host institution shall:

13.2.1. Ensure that the study design is scientific and ethical.

13.2.2. Ensure ethical implementation of the research.

13.2.3. Comply with legal requirements and ethics regulations as stipulated in this guideline.

13.2.4. Ensure that the investigators conducting the study are scientifically qualified and com petent to carry out the research at the institution.

13.2.5. Facilitate and provide support for smooth and ethical implementation of the research.

13.2.6. Make sure that the results of the study are properly and publicly disseminated.

13.2.7. Ensure that guidelines, ethical principles, and related materials reach the end users and the investigators.

13.2.8. Provide periodical reports of ethical implementation of the study to the IRB's Secretariat.

13.2.9. Take disciplinary action on the investigators for breach of any of this guidelines, regulatory and legal requirements.

13.3 Sponsors

Sponsors/Donors are responsible for providing an environment that promotes integrity, objectivity and the highest ethical standards of research, including standards for design, implementation and reporting. Particularly, sponsors must commit to protect participants in all research. Besides, sponsors are expected to ensure research subjects and communities are not made worse off during or after completion of the research. Sponsors can accomplish these goals in the following ways:

13.3.1. Ensure appropriate review and approval by appropriate IRBs. If the sponsor is based outside of Ethiopia, the sponsor must in addition produce approval from an appropriate IRB where the sponsor is based. If the sponsor is an international organization, the review of protocol must maintain rigor in accordance with its own independent IRB.

13.3.2. Monitor the research according to a plan approved by the IRB.

13.3.3. Select qualified investigators and institutions as collaborators, sponsors.

13.3.4. Provide ethical guidelines to all investigators.

13.3.5. Complying with the local ethical, regulatory and legal requirments.

13.3.6. Promoting research integrity.

13.3.7. Ensuring the local relevance of the research by involving local partners in the developmental stages.

13.3.8. Financing the study.

13.3.9. Ensuring adequate safety and efficacy of investigational products, if applicable.

13.3.10. Ensuring safety and efficacy of investigational products, if applicable; the sponsor shall ensure investigational products are manufactured following good manufacturing practice,

13.3.11. Supplying and handling investigational products.

13.3.12. Updating investigators' brochure as significant new information is made available.

13.3.13. Establishing a DSMB, if applicable.

13.3.14. Assigning clinical monitors, if applicable.

13.3.15. Providing insurance to study participants in case of injury.

13.3.16. Providing special forms for recording and reporting of serious adverse events and ensure adverse events are appropriately investigated and managed until resolution or stabilization.

13.3.17. Informing the IRB if it suspends or terminates a research with detailed explanation for the termination or suspension.

13.3.18. Ensuring the community where the research is conducted is informed about the research findings.

13.4 Donors

Donors are responsible for providing an environment that promotes integrity, objectivity and the highest ethical standards of research. Donors can accomplish these goals in the following ways:

13.4.1. Ensure appropriate review and approval by appropriate IRBs.

13.4.2. Monitor the research according to a plan approved by the IRB.

13.4.3. Complying with the local ethical, regulatory and legal requirements.

13.4.4. Promoting research integrity.

13.4.5. Ensuring the local relevance of the research by involving local partners in the developmental stages.

13.4.6. Financing the study.

13.4.7. Providing insurance to study participants in case of injury.

13.4.8. Informing the IRB if it suspends or terminates the financial support.

13.4.9. Ensuring the community where the research is conducted is informed about the research findings.

14. Networking

Networking and creating a dynamic relationship among IRBs is essential for:

- Establishing a strong data base system to standardize and streamline set of data elements.
- Facilitating coordination among IRBs.
- Establishing a strong and standard ethical review system within Ethiopia and harmonize safety reporting.
- Building capacity and strengthening partnership among IRBs.
- Sharing of experiences among local and international IRBs.
- Establishing a central web-based repository.

14.1 Networking Mechanisms/Modalities

14.1.1. Information flow should be multidirectional.

14.1.2. All IRBs shall submit reports to NRERC annually.

14.1.3. IRBs in federal institutions shall send their biannual reports directly to the NRERC.

14.1.4. Reports of the respective IRBs shall include at a minimum: activities performed, support needed, problems encountered, etc.

14.1.5. For urgent matters, IRBs should seek information or technical help from NRERC at any time through its Secretariat.

14.1.6. The NRERC will distribute appropriate guidelines and other related information to other IRBs and request feedback.

14.1.7. The NRERC establishes working relations with local and international IRBs through its Secretariat.

14.1.8. Reporting at each level should be by mail, fax or email.

Bibliography

1. The Constitution of The Federal Democratic Republic of Ethiopia. Addis Ababa, August 21 1995.
2. Federal Negarit Gazetta of The Federal Democratic Republic of Ethiopia. 15th Year No 1 Addis Ababa, October 24 2008.
3. Federal Negarit Gazetta of The Federal Democratic Republic of Ethiopia. 16th Year No 51 Addis Ababa, August 23 2010.
4. Ethiopian Science and Technology Commission. National Health Research Ethics Review Guidelines, Fourth Edition. Addis Ababa, Ethiopia 2005.
5. Guidelines for Conducting Clinical Trials. Drug Administration and Control Authority. Addis Ababa, Ethiopia.
6. International Ethical Guidelines for Biomedical Research Involving Human Subjects. Council for International Organizations of Medical Sciences (CIOMS) in collaboration with the World Health Organization (WHO). CIOMS Geneva 2002.
7. Code of Federal Regulations. TITLE 45 — PUBLIC WELFARE. Department of Health and Human Services. PART 46: PROTECTION OF HUMAN SUBJECTS. Revised June 23, 2005. Effective June 23, 2005.
8. The Belmont Report. Ethical Principles and Guidelines for the Protection of Human Subjects of Research. The National Commission for the Protection of Human Subjects of Biomedical and Behavioral Research. April 18, 1979.
9. Nuremberg Code. 1948.
10. World Medical Association Declaration of Helsinki. Ethical Principles for Medical Research Involving Human Subjects. Adopted by the 18th WMA General Assembly. Helsinki, Finland, June 1964, and amendments in 1975, 1983, 1989, 1996, 2000. Note of Clarification on Paragraph 29 added in 2002. Note of Clarification on Paragraph 30 added in 2008.
11. Karbwang J, Pattou C. Standard operating procedures for clinical investigators. UNDP/World Bank/WHO, special program for research and training in tropical diseases (TDR). TDR/TDP/SOP/99.1.
12. Operational Guidelines for Ethics Committees that Review Biomedical Research. WHO, 2000.
13. International Declaration on Human Genetic Data, UNESCO, 2003.
14. Operational Guidelines for the Establishment and Functioning of Data and Safety Monitoring Boards, TDR-WHO, Geneva 2005.
15. Uganda National Council for Science and Technology, 2007. National Guidelines for Research involving Humans as Research Participants. Kampala – Uganda: UNCST.

16. Statement of WHO Expert Advisory Group on Ethical issues in Medical genetics. WHO, Geneva 1998.
17. Ethical Guidelines for International Comparative Social Science Research in the framework of Management of Social Transformations, MOST. UNESCO, MOST Paris.
18. Hyder A, Dowson L, Bachani AM, Lavery J. Moving from research ethics review o research ethics systems in low-income and middle-income countries. Lancet 2009;37:862-65.
19. Heath EJ. The IRB's Monitoring Function: Four Concepts of Monitoring. IRB: Ethics and *Human Research*, 1979; 1: 1-3+12.
20. DeMets DL, Fost N, Powers M. An Institutional Review Board dilemma: responsible for safety monitoring but not in control. *Clin Trials* 2006; 3; 142.
21. Cowan DH. Scientific Design, Ethics, and Monitoring: IRB Review of Randomized Clinical Trials. IRB: *Ethics and Human Research*, 1980;2: 1-4.
22. Karlawish, Hall *(1996)*. Am J Respir Crit Care Med, 1996;153:499-5-6.
23. Casarett, Karlawish, Sugarman. JAMA, 2000, 283 (17): 2275-80.
24. Richards HM, Schwartz LJ. Ethics of qualitative research: are there special issues for health services research? Family Practice 2002; 19: 135-139.

Appendix I. Acronyms

AAUMFIRB	Addis Ababa University Medical Faculty Institutional Review Board
AE	Adverse Event
AHRI	Armauer Hansen Research Institute
CAB	Community Advisory Board
CIOMS	Council for International Organization of Medical Sciences
COI	Conflict Of Interest
CV	Curriculum Vitae
DACA	Drug Administration and Control Authority
DNA	Deoxyribo Nucleic Acid
DSMB	Data and Safety Monitoring Board
EC	Ethics Committee
EHNRI	Ethiopian Health and Nutrition Research Institute
EPHA	Ethiopian Public Health Association
ERC	Ethics Review Committee
ESTA	Ethiopian Science and Technology Agency
ESTC	Ethiopian Science and Technology Commission
FDRE	Federal Democratic Republic of Ethiopia
FMHCACA	Food, Medicine and Health Care Administration and Control Authority
GDP	Gross Domestic Product
GCP	Good Clinical Practice
GMP	Good Manufacturing Practice
GNI	Gross National Income
RE	Research Ethics
NRERCs	National Research Ethics Review Committees
HS&T	Health Science and Technology
IBC	Institutional Bio-safety Committee
IRB	Institutional Review Board
LAR	Legally Authorized Representative
MOST	Ministry of Science and Technology
MTA	Materials Transfer Agreement

NEC	National Ethics Committee
NGO	Non-Governmental Organization
PI(s)	Principle Investigator(s)
SAE	Serious Adverse Event
SOP	Standard Operating Procedures
S&T	Science & Technology
TOR	Terms of Reference
UN	United Nations
WHO	World Health Organization

Appendix II. Glossary of Terms

Accreditation-The act of granting credit or recognition for meeting established standards; a process to ensure quality and stimulate continuing improvement.

Adverse Event (AE)-Any untoward health-related occurrence in a participant administered a health-research intervention and which does not necessarily have to have a causal relationship with this intervention. An AE can be any unfavorable and unintended sign, symptom, or condition temporally associated with the administration of the health-research intervention, whether or not considered related to the intervention. This also includes unfavorable deviations from baseline health.

Amendment-A material change to study procedures. Such changes, including minor changes, must be reviewed by an IRB before they may be implemented.

Anonymous/anonymized-Lacking identification because identifiers or other information that could identify the individual were not collected or were removed. Information may or may not be considered anonymous if there is a reasonable basis to believe that one can use the information to identify and individual, even if one cannot readily ascertain the individual's identity; see further discussion at individually identifiable information. For example, it might be possible to determine a survey respondent's identity from a combination of demographic factors in a survey together with public or proprietary data sources, even if the surveyor did not jointly record the respondent's identity and those demographic characteristics..

Approve/approval-The determination of the IRB that the research has been reviewed and may be conducted at an institution within the constraints set forth by the IRB and by other institutional and federal requirements.

Assent-Participant *not* of legal age (i.e., child); affirmative agreement to participate in research. Mere failure to object should not be construed as assent.

Autonomy-Self-rule that is free from both controlling interference by others and from limitations that prevent meaningful choice. See also respect for persons.

Belmont Report-Statement of ethical principles and guidelines for the protection of

human participants of research. The National Commission for the Protection of Human Subjects of Biomedical and Behavioral Research published this report in 1979. The three principles are respect for persons, beneficence, and justice. See: https://www.hhs.gov/orhp[/humansubjects/guidance/belmont.htm

Beneficence, principle of-Ethical precept asserting an obligation to prevent harm, to remove harm, or to do or promote good; two-part rule: (1) do not harm and (2) maximize possible benefits and minimize possible harms.

Benefit-A potential advantage or gain.

Breach of protocol-Material departure from approved procedures of the study, such as the consent process, violations of data confidentiality, or complaints by participants; may be a reportable incident.

Child/children-Person who has not attained the legal age for consent to treatments or procedures involved in the research, under the Ethiopian law

Clinical trial-A prospective research study in human participants that is designed to answer specific questions about health-related interventions (such as medications, herbal supplements, nutritional strategies, physical interventions, behavioral interventions, prevention trials, or diagnostic tools), particularly to determine whether these interventions are safe, efficacious, and effective.

Close/closure-Proactively and permanently end both research-related intervention or interaction with participants and collection and use of identifiable private research information when study objectives have been met as specified in the protocol.

Coded-Replacement of identifying information (such as name or social security number that would enable one to readily ascertain the identity of the individual to whom the private information or specimens pertain) with a number, letter, symbol, or combination thereof where a key to decipher the code exists and links the identifying information to the private information or specimens. In contrast with linked, coded often means that the link exists but is unavailable, as through a nondisclosure arrangement.

Compensation-Payment to cover actual research-related harm. Where understandability is an issue, use a simpler word like “payment”. Contrast with incentive, reimbursement.

Completion of study-Point at which data analysis has ended or identifying information is removed from the data and biological specimens. See also close.

Comprehension–in this context, understanding what the study is about.

Conduct-To be engaged (in research) by obtaining data about living individuals through intervention or interaction with them for research purposes or by obtaining individually identifiable private information about living individuals for research purposes.

Conflict of interest-A situation where the goals or obligations of an investigator or reviewer conflict with an obligation to uphold another party’s interest, thereby compromising objectivity and impartiality.

Confidential/confidentiality-The condition of honoring a request or expectation that information will be protected from disclosure.

Convened-A formal, joint meeting or action of a quorum of the IRB.

Collaborative research-Research that involves more than one institution.

Council for International Organizations of Medical Sciences (CIOMS)-An international, non-governmental, non-profit organization established in 1949 whose membership represents many of the biomedical disciplines and national academies of sciences and medical research councils. A main objective of CIOMS is to facilitate and promote international activities in the field of biomedical sciences, especially when the participation of several international associations and national institutions is deemed necessary.

Data and safety monitoring-Structured, ongoing monitoring of specified characteristics of a research protocol, generally by a small, independent body of experts appointed by the study sponsor. Sometimes incorrectly called "data safety and monitoring". Monitoring may pertain to study performance (such as rate of accrual), safety (such as occurrence of AEs), and efficacy (such as achievement of primary endpoints). A body that monitors all three characteristics is usually called a data monitoring committee (DMC) or a data and safety monitoring board (DSMB).

Declaration of Helsinki-A statement of ethical principles to provide guidance to physicians and other participants in medical research involving human participants; adopted by the World Medical Assembly in 1964 and last updated in 2004. See http://www.wma.net/e/policy/b3.htm.

Disapprove/disapproval-The determination by the IRB that the research may not be conducted at an institution

Documentation of consent-Consent that is documented by the use of a written consent form approved by the IRB and signed by the participant or the participant's legally authorized representative.

Emergency response-A public health activity undertaken in an urgent or emergency situation, usually because of an identified or suspected imminent health threat to the population, but sometimes because the public or government authorities perceive an imminent threat that demands immediate action. The primary purpose of the activity is to document the existence and magnitude of a public health problem in the community and to implement appropriate measures to address the problem (Langmuir, 1980).

Exemption/exempt research-Categories of research to which the Federal regulations for human research protections do not apply. Research involving prisoners and some research involving children are not exempt.

Expected-Pertaining to an AE, the event has been previously observed or documented in humans under the research intervention (or one substantially similar), and the nature or severity of the event is consistent with information in the relevant source documents (e.g., investigator's brochure, package insert, or non-reportable events [NRE] list).

Expedited review-Review performed by the IRB Chair or a designated experienced member for research that involves no more than minimal risk and meets the criteria for expedited review or represents minor changes in approved research.

Experienced reviewer -A member of an IRB who is designated by the Chair as authorized to conduct expedited reviews.

Expert reviewer-A member of an IRB who is designated by the Chair as authorized to conduct expedited reviews.

Expiration date-The expiration date is the day before the anniversary of the approval date – unless the IRB approves it for less than one year. For instance, a protocol approved on 12/4 has approval date of 12/4/06 and an expiration date of 12/3/07 midnight (which means research may be conducted on 12/3).

Fetus-The product of conception from the time of implantation until delivery.

Focus group-Group of individuals brought together to discuss an issue within a structured environment, often for formative or qualitative research purposes.

Generalizable knowledge-New information that has relevance beyond the population or program from which it was collected

Good Clinical Practice (GCP)-An international ethical and scientific quality standard for designing, conducting, recording, and reporting trials that involve the participation of human participants. See also http://www.fda.gov/oc/gcp/default.htm.

Guardian-An individual who is authorized under applicable local law to consent on behalf of a child to general medical care.

Harm-Injury, damage, or hurt; an experience in which one's interests are thwarted, defeated, or set back, especially one's physical or psychological interests.

Human subject/human participant-A living person about whom an investigator conducting research obtains (1) data through intervention or interaction with the individual, or (2) identifiable private information (e.g., medical records, employment records, or school records).

Identifiable private information-Information (data or biological specimens) such that the identity of a participant is or may readily be ascertained by the investigator or associated with the information, and either the information concerns behavior that occurs in a context in which an individual can reasonably expect that no observation or recording is taking place, or the information has been provided for specific purposes by an individual and which the individual can reasonably expect will not be made public.

Incentive-Payment or other goods or services offered to motivate study participation.

Incident-An instance of one of the following: an unanticipated problem involving risks to participants or others, serious or continuing noncompliance, or suspension or termination (for reasons other than expiration).

Informed consent-The free and informed decision by a prospective participant or

participant's legally authorized representative to participate in research. The consent process should ensure that the participant has been provided full information regarding the research, the participant comprehends the research, and the participant is volunteering free of coercion and undue influence.

Injury-Physical harm to a participant in a research study.

Institutional Review Board (IRB)-The formally appointed ethics review committee established to ensure that research involving human participants conforms to ethical principles

Intervention-Physical procedures by which data are gathered (e.g., venipuncture) and manipulations of the participant or the participant's environment that are performed for research purposes.

Justice, principle of-Ethical precept asserting an obligation to treat persons fairly and give to each person what she is due; two-part rule: (1) exhibit fairness and (2) distinguish between classes of participants that ought, and ought not, to participate in any particular kind of research.

Legally Authorized Representative (LAR)-An individual or judicial or other body authorized under applicable State law who may consent on behalf of another individual to participate in the procedure(s) involved in the research.

Local IRB-IRB located in the institution where the research is to be conducted.

Medical device-Any instrument, apparatus, or other similar or related article, including component, part, or accessory, which is (a) recognized in the official National Formulary, or the United States Pharmacopeia, or any supplement to them; (b) intended for use in the diagnosis of disease or other conditions, or in the cure, mitigation, treatment, or prevention of disease, in humans or other animals; or (c) intended to affect the structure or any function of the human body or in animals; and does not achieve any of its principal intended purposes through chemical action within or on the human body or in animals and is not dependent upon being metabolized for the achievement of its principal intended purposes.

Minimal risk-(a) Risk such that the probability and magnitude of harm or discomfort anticipated in the research are not greater in and of themselves than those ordinarily encountered in daily life or during the performance of routine physical or psychological examinations or tests.

Minor changes-Changes to a research protocol that do not result in a net increase in risk, a change in the harm-benefit balance, or provide minor clarification or correction. Minor changes may be reviewed under expedited review, even if the protocol has been deemed to pose more than minimal risk to participants.

Noncompliance-Failure by investigators, research staff, IRB members, or IRB staff to follow regulations for human research protections

Oral consent-Consent obtained only through speaking, generally in the absence of documentation of consent. See also documentation of consent, informed consent, verbal consent.

Parental permission-The agreement of a parent or guardian to the participation of their child in research.

Personal identifier-Information obtained and recorded in such a manner that human participants can be recognized, directly or through links to the participants. Examples include names, social security numbers, and codes.

Pilot study-Preliminary study to determine the feasibility of a larger study, use of a test instrument, or other activity.

Pregnancy-The period of time from implantation until delivery. A woman shall be assumed to be pregnant if she exhibits any of the pertinent presumptive signs of pregnancy, such as missed menses, until the results of a pregnancy test are negative or until delivery.

Principal Investigator (PI)-Lead scientist who is working on the design of a research study, development of methods and procedures for the study, collection of data or specimens, analysis of data or specimens, or interpretation of data.

Prisoner-Any individual involuntarily confined or detained in a penal institution. The term is intended to encompass individuals sentenced to such an institution under a criminal or civil statute, individuals detained in other facilities by virtue of statutes or commitment procedures which provide alternatives to criminal prosecution or incarceration in a penal institution, and individuals detained pending arraignment, trial, or sentencing.

Private information-information about individually identifiable behavior that occurs in a context in which the individual can reasonably expect that no observation or recording is taking place, and information which has been provided for specific purposes by an individual and which the individual can reasonably expect will not be made public (e.g., a medical record).

Private/privacy-Individual person's interest in preventing disclosure of information about himself or herself.

Project-A planned activity or collection of activities conducted for a particular purpose; may encompass more than one protocol on the same subject matter.

Protocol-The formal design or plan of a data collection activity; specifically, the plan submitted to a reviewing authority such as an IRB. The protocol includes a description of the design or methods for conducting the data collection, description of the study population, methods for data handling and analysis, procedures for handling incidents, and methods for notification and dissemination of results.

Quorum-The number of IRB members required to be present at a convened meeting in order for the IRB to transact business.

Reimbursement-Repayment for costs incurred by virtue or participation in research, such as for lost earnings or travel costs.

Related/relation-Pertaining to an AE, the likelihood that the event was caused by research procedures, usually relative to the likelihood it was caused by something

other than research procedures.

Report-A written account of the IRB's findings, conditions for approval, or reasons for disapproval regarding human research protections in a research protocol.

Repository-An entity that collects, stores and manages, and distributes data or human tissue materials to recipient investigators for research and other purposes. See also http://www.hhs.gov/ohrp/humansubjects/guidance/reposit.htm.

Research-Systematic investigation, including development, testing, and evaluation, designed to develop or contribute to generalizable knowledge.

Respect for persons, principle of-The requirement to treat individuals as autonomous agents and to provide additional protections to persons with diminished autonomy. Respect for persons requires that participants, to the degree that they are capable, be given the opportunity to choose what shall or shall not happen to them.

Response-An investigator's written reply to an IRB report.

Responsible conduct of research (RCR)-A collection of core areas for conducting scientific research with integrity: data acquisition, management, sharing and ownership; conflict of interest and commitment; human participants; animal welfare; research misconduct; publication practices and responsible authorship; mentor/trainee responsibilities; peer review; and collaborative science

Risk-Exposure to injury, loss, or harm, expressed in terms of the probability and magnitude of that harm. Risks to participants must be minimized and must be reasonable in relation to anticipated benefits to participants and the importance the knowledge that may reasonably be expected to result.

Serious adverse event (SAE)-An AE that results in death, is life-threatening (at the time of the event), requires inpatient hospitalization or prolongation of existing hospitalization, results in persistent or significant disability or incapacity, or is a congenital anomaly or birth defect. An AE may also be considered serious if it jeopardizes the participant or requires intervention to prevent one of the other outcomes listed.

Serious noncompliance-Noncompliance that results in increased risk to participants or reflects a failure to apply substantial portions of governing regulations. Serious noncompliance must be reported promptly.

Severe/severity-The graded level of intensity of an AE and its interference with usual social and functional activities, often standardized in toxicity tables. Grade levels generally include normal, mild, moderate, severe, life-threatening, and fatal.

Sponsor- a person or other entity that initiates a clinical investigation, but that does not actually conduct the investigation.

Suspend/suspension-Temporary cessation of research-related intervention or interaction with participants and obtaining or using identifiable private research information. Partial suspension halts some but not all such activities, for example when enrollment is stopped but follow-up continues with enrolled participants. A suspension must be

reported promptly unless the suspension results from expiration of IRB approval.

Terminate/termination-Permanent cessation of research-related intervention or interaction with participants and obtaining and use of identifiable private research information. A termination must be reported promptly unless the termination results from expiration of IRB approval or withdrawal or closure for reasons other than research risks.

Understandable language-generally 8th grade reading level or lower/higher depending on targeted population.

Undue influence-An excessive, unwarranted, inappropriate, or improper reward intended to motivate study participation.

Unexpected-Pertaining to an AE, the event is previously unobserved or undocumented in humans under the research intervention (or one substantially similar), the nature or severity of the event is not consistent with information in the relevant source documents (e.g., investigator's brochure, package insert, or non-reportable events [NRE] list), or the event is observed with higher frequency than previously observed or documented. Expectedness does not entail the ability to predict results from in vitro, animal, or other pharmacological models.

Unlinked-The condition of data or specimens which had been coded but for which the key linking the code to direct personal identifiers has been destroyed.

Unrelated-Pertaining to an AE, the condition in which the event is due to a documented cause other than research procedures.

Verbal consent-Consent obtained by communication in words; this may include spoken or written format.

Voluntary/voluntariness-Freedom from coercion and undue influence

Vulnerable-Having reduced capacity to offer free and informed consent due to possible coercion, undue influence, or other diminished autonomy, such as children, prisoners, pregnant women, mentally disabled persons, or economically or educationally disadvantaged persons as vulnerable. Vulnerability may be associated with other characteristics such as age, health status, or social standing. The IRB must ensure additional safeguards protect the rights and welfare of vulnerable persons.

Vulnerable population-A group identified by one or more common characteristics associated with reduced capacity to offer free and informed consent.

Waive/waiver-Temporarily set aside the requirement of a particular rule, regulation, or condition in a protocol or consent document.

Withdraw/withdrawal-Permanently halt a research study after submission for IRB review but before human participants become involved, whether or not the study has been reviewed by an IRB. This term has been replaced by the term close/closure.

Appendix III: NRERC Terms of Reference

1. Membership

The Ministry of Science and Technology designates fifteen persons to be members of the NRERC; members can also be co-opted as deemed necessary. To ensure adequate review of proposals, the members will be professionals from different academic backgrounds such as clinical, biomedical, public health, law, and behavioral sciences. As much as possible membership shall be gender sensitive and ensure layperson representation. The committee shall elect its chairperson and Vice chair person.The director General of MoST shall assign non voting secretary to the NRERC from MoST.

2. Tenure

The tenure of the NRERC shall be three years.

3. Replacement of Missing Members

Whenever a member is absent for three consecutive meetings without valid reasons, the Ministry will be notified of his/her absence and requested for replacement.

4. Meetings

The Committee meets every two months regularly. The Chairman however, in consultation with the Secretariat, may call an extra ordinary meeting at any time if deemed necessary.

5. Quorum

The quorum will consist of eight (or >50%) members. In the absence of the chairperson, temporary chairperson shall be elected.

6. Votes

Decisions will be made on a consensus basis. Whenever there is a difference among committee members that cannot be resolved by discussion, decisions will be made by majority votes; all members have equal voting rights. When votes are at par, the decision the chairperson supports will be final.

7. Deadline for ethics review

Every project proposal will get cleared by this Committee with a maximum period of three months from the time the NRERC seats to review the protocol.

8. Review procedures

Upon receiving the proposal, the secretariat shall circulate the proposal among NRERC members for evaluation using the Ethics Review Form. Opinions of the committee members will be sought during the regular meetings and minutes will be taken on the final decision and communicated to the applying institution and the Principal Investigator should be copied through the Secretariat. All minutes shall be signed by both the Chairperson and Secretary.

9. Information to Investigators

In order to save time, all the necessary requirements for the Ethics review will be provided to investigators in advance along with the National Ethics Review Form.

10. Ethics of the NRERC

Documents that reach the Committee will be strictly confidential.

11. Monitoring & Evaluation

The NRERC shall ensure the establishment of a Data Safety and Monitoring Board for all clinical trials. It also evaluates the ethical implementation of projects by conducting site visits, assessing progress reports, and/or consulting study participants.

12. Legal Issues

The NRERC presents to the MoST all legal issues related to breach of health research ethics for appropriate action.

Appendix IV: National Research Ethics Review Application Form

Fill in the spaces and mark (X) wherever appropriate

A. General

1. Title of the project
2. Name and address of the principal investigator(s)
3. Name and address of the applying institution(s)
4. Specific request of the application
   - a)Ethics Review ☐
   - b)Renewal (extension) ☐
   - c)Termination ☐
   - d)Resubmission ☐
   - e)Specify if any other ☐

B. Specifics

5. Number of submitted copies
6. Number of submitted pages excluding all attachments
7. Number of submitted pages including all attachments
8. Required annexes/attachments
   - 8.1. Written informed consent form ☐
   - 8.2. Information sheet ☐
   - 8.3. Officially endorsed minutes of IRB ☐
   - 8.4. Letter(s) of collaboration from all collaborating institution(s) ☐
   - 8.5. Ethics review letter from collaborating and host institution(s) ☐
   - 8.6. CVs of the PI and relevant senior co- investigator(s) ☐
   - 8.7. Material transfer agreement (MTA) ☐
   - 8.8. Specify if any other ☐
9. Name and signature of applying PI and host institution (s) as an expression of accountability, responsibility and authenticity of the request and prior commitment of ethical implementation of the research after approval.

______________	______________	______________
Name (PI)	Signature	Date
______________	______________	______________
Name (Host, institution)	Signature (Head)	Date

Seal and stamp

Appendix V. Research Ethics Review Form

Research Ethics Review Form

Title: ____

Reviewer Name: ____________________________________

Criteria/Item	Rating
1. Consent Form<br>• Does the consent form contain all the necessary information that the subject should be aware of?	_____Yes<br>_____Requires revision<br>_____No<br>_____Not applicable<br>_____Not attached
2. Are the objectives of the study clearly stated?	_____Yes<br>_____No
3. Are the methods ethically sound?<br>(in respect to Beneficence, Justice and Respect for Person)	_____Yes<br>_____Not well described<br>_____No
4. Are provisions to overcome risks well described and acceptable?<br>• DSMC	_____Yes<br>_____No<br>_____Not applicable
5. Are there provisions to provide standard/best proven care?	_____Yes<br>_____No<br>_____Not applicable
6. Are the safety procedures in the use of vaccines, drugs and other biological products acceptable?	_____Yes<br>_____No<br>_____Not applicable
7. Are the procedures to keep confidentiality well described?	_____Yes<br>_____No<br>_____Not applicable
8. Are the proposed researchers competent to carry out the study in a scientifically sound manner?	_____Yes<br>_____No<br>_____Not applicable<br>_____Unable to access
9. Does it have material Transfer Agreement?	_____Yes<br>_____No<br>_____Not applicable
Recommendation	____Approved_____Approved on Conditions _____ Not Approved
Remarks (If any)	

(Please use additional paper if you comments to forward)

Appendix VI. Material Transfer Agreement Form

Ministry of Science and Technology, Federal Democratic Republic of Ethiopia

National Research Ethics Review Committee

Address: Tel.: +251011-4-674353 P.O. Box: 2490 Fax: +251-011-4-660241

e-mail:

Addis Ababa –Ethiopia

Annex #: Material Transfer Agreement

This Material Transfer Agreement (MTA) has been prepared for use by ___________ and __________ in all transfer of research material (samples, derivatives, and specimens) related to the protocol. ____

Provider: ______________________________________

Recipient: ______________________________________

1. Provider agrees to transfer to recipient's designated (__________) the following research materials /specimen. ____

   ____

   The research material will only be used for research purposes as described in the protocol by recipient's investigator in designated laboratory for the research project described below, under suitable containment conditions. This research material will not be used for commercial purposes such as screening, production or sale for which a commercialization license may be required. Recipient agrees to comply with all National and International guidelines rules and regulations applicable to the Research Project and the handling of the Research Material.

   a) Are the research materials of human origin?

      Yes ☐ No ☐

   b) If yes, are they collected according to the details in the protocol and in adherence to National Research Ethics Review Committee (NRERC) and ______________ Ethics Review Committee recommendations and their approval.

      Yes ☐ No ☐

2. This research material and its derivatives will be used by recipient's investigator solely in connection with the following research project ("Research Project") de-

scribed with specificity as follows ____________________________________

____

3. In all presentations or written publications concerning the research project, recipient will seek agreement of provider and acknowledge provider's contribution of this research material unless requested otherwise.
4. This research material represents a significant contribution on the part of provider and is considered proprietary to provider. Recipient therefore agrees to retain control over this research Material and further agrees not to transfer the research material to other people not under her/his direct supervision with out advance written approval of provider. The research material will be disposed of as agreed upon per protocol at the end of completion of the project on ____________.
5. The provider does not take any responsibility for loss, damage, wastage or spoilage of the research material during or after shipment to the address provided by the recipient under conditions agreed to in the protocol on shipment of the samples. This research material is provided as a service to the research community. IT IS BEING SUPPLIED TO RECIPIANT WITH NO WARRANTIES, EXPRESS OR IMPLIED, INCLUDING ANY WARRANTY OF MERCHANTABILITY OR FITNESS FOR A PARTICULAR PURPOSE. Provider makes no representations that the use of the research material will not infringe any patent or proprietary right of third parties.
6. The recipient shall notify the provider in witting of any intention, improvement, modification discovery or development to the material or the information made by recipient or parties, collaborating with recipient, here in after referred to as "invention". Nothing in this agreement shall, however, be construed as conveying to the provider any rights under any patents or other intellectual property to such invention, other than as explicitly provided herein. At its option the provider shall be entitled to receive sample of any materials derived from the Materials for its own research and evaluation purposes only.
7. The under- signed provider and recipient expressly certify and affirm that the contents of any statements made here in are truthful and accurate.
8. Any additional terms (use an attached page if necessary):
9. The provider maintains, ownership right of the research material and its derivatives unless stated otherwise.

The provider will retain a copy (aliquot) of every sample sent abroad as much as possible for local research needs.

Material Transfer Agreement

Signature page

For Recipient:

Recipient's Investigator

Signature

Date ---------------

Mailing Address for Material:

Tel: _______________

Fax: _______________

Duly Authorized

Signature/ Stamp

Date ----------------

Mailing Address for Notices:

Tel: _______________

Fax: _______________

For Provider

Provider's Investigator

____

Signature

Date _______________

Mailing Address :

P.o.Box ___________

Tel: ___________

Fax: ___________

Duly Authorized

____

Signature

Date ______________

Mailing Address for Notices:

P.o.Box ___________

Tel: ___________

Fax: ___________

Appendix VII. List of Participants to enrich NRERC Guideline

1. National Consultative Workshop To Finalize the National Research Ethics Review Guideline

December 1-2, 2011, Gion hotel, Addis Ababa

No	Name	Organization
1.	Prof.Berhanu Erko	NRERC Chair Person /Aklilu Lema Institute of Pathobiology
2.	prof. Beyene Petros	AAU, Collage of Natural Science
3.	Ms. Fran Sanden	CDC
4.	prof. Melakeberhane Dagne	AAU, Collage of Health Science
5.	prof.Asrat Hailu	AAU, Collage of Health Science
6.	Dr .Alemayehu Mekonnen	CDC
7.	Dr Getnet Yimer	AAU, Collage of Health Science
8.	Mr .Solomon Shiferaw	FMHACA
9.	Mr. Yonas Girmay	FMHACA
10.	Mr. Abrham G/giorgis	WHO
11.	Dr. Solomon Kumbi	NRERC Vic. Chairperson/ AAU, Collage of Health Science
12.	Mr. Hailegnaw Eshete	EPHA
13.	Prof. Yemane Berhane	ACIPH
14.	Dr.Yemane Teklaye	Consultant
15.	Prof.Yeweyenhareg Feleke	AAU/EMA
16.	Dr. Yemtubezinah W/A	AAU, Collage of Health Science
17.	Miluet tamirat	AAU, Collage of Health Science
18.	Abraham Tarekegn	AAU, Collage of Health Science
19.	Dr Eshetu Lema	EHNRI
20.	Dr Belete Tegberu	EHNRI
21.	Dr Zelke W/Tensge	Ethiopian Biodiversity institute
22.	Dr Wubjege Tshome	AAU, Collage of Health Science
23.	Meka Metkiyqa	Head, health research dep.
24.	Tadesse Bekele	Harmaye university
25.	Wendemu Gebeyehu	Amhara Regional Health Bureau
26.	Wendemu Ashenafi	Harmaye university
27.	Dr Adrajew Amsalu	Hawassa Universty
28.	Dr Tamerat Degafu	National Veterinary Institute

29.	Tesfaye Lema	National Veterinary Institute
30.	Afework Mulugeta (Dr)	Mekelle University
31.	Mulu Adere	Gondar University
32.	Mekonnen Hailsilas	Mekelle University
33.	Hassen Mowhid	Somalia regional Health bureau
34.	Shetaye Alemu (Dr)	Gondar University
35.	Yared Merid	Hawassa University
36.	Beyene Wondafrash	Jima University
37.	Alemseged Abdissa	Jima University
38.	Dr. Ermias Diro	Gondar University
39.	Dr .Daniel Girma	Miz-hasab R.C
40.	Tujuma Guta	Oromia health bureau
41.	Girma Desta	EPHA
42.	Kebede Abera	AAU
43.	Yared Awugechew	MoST, NRERC Secretary
44.	Germa Yosef	MoST
45.	Mustefa Abdulsemed	MoST
46.	MuluGegta Wube	MoST
47.	Yohannes Sitotaw	MoST

2. Validation workshop to finalize National research Ethics Review Guideline, October 2,2013,Dreamland Hotel, Bisoftu

No	Name	Organization
1.	Prof. Demesie Habte	Ethiopian Science Academy
2.	Dr Abraham Aseffa	AHRI
3.	Prof. Endashaw Bekele	AAU, college of Natural Science
4.	Dr Geremu Tarkegne	ALERT, Training Center
5.	Dr Hassen Mamo	AAU, College of Natural Science
6.	Dr Mekonen Hailsilase	Tigray Science and Technology Agency
7.	Dr Belayneh Admasu,	Biotechnology research Institute ,Ministry of Agriculture
8.	Dr Ermias Diro	University of Gondar

9.	Mr Techalew Schemels	Hawasa University
10.	Prof. Yeweyenhareg Felek	AAU, College of Health Science
11.	Prof. Melakberhane Dagne	AAU, College of Health Science
12.	Dr. Getnet Yemer	AAU, College of Health Science
13.	Dr Efrem Engedawerk,	AAU, College of Health Science
14.	Dr .Yimtubezenash W/Aregaye	AAU, College of Health Science
15.	Prof. Sleshie Lulseged	ICAP
16.	Dr Mengestu Leges	AAU, College of Social Science, School of Social work
17.	Dr Adamu Adessie	Addis Ababa University, College of Health Science
18.	Dr Mengistu Legesse	AAU, Aklilu Lema Patobiology
19.	Dr Degu Jerene	JHU
20.	Dr Esayas	National Veterinary Institute
21.	Mr Araya feseha	Ethiopian National Accreditation office, Addis Ababa
22.	Ms Wubjege Teshome	FMHACA
23.	Prof. Brihanu Erko	NRERC, Chairperson
24.	Dr Solomon Kumbi,	NRER, Vice chairperson
25.	Dr Workenesh Ayele	NRERC ,Member
26.	Mr Yohannes Sitotaw	NRERC Secretary
27.	Dr Liya Wassie	NRERC, Member
28.	Mr Geberemariam Birhanu	NRERC, Member
29.	Mr.Teshome Sahelemariam,	MoST
30.	Mr Girma Yosef,	MoST
31.	Mr Getachew Atente,	MoST
32.	Mr. Abdisa Yilma	MoST
33.	Mr. Babesha Kenaw	MoST

34.	Mr Melaku Arage	MoST
35.	Mr Sisay Gardew	MoST
36.	Mr Hailegnaw Eshete,	EPHA
37.	Mr Derje Syium	EPHA
38.	Mrs. wudenshe Mamo	EPHA
39.	Mr Mustefa Abdulsemed	MoST
40.	Mr. Mulugeta wube	MoST
41.	Mrs. Serkalem Ketema	MoST
42.	Mr Shibeshi Kasa	MoST
43.	Mr. Solomon Getachew	MoST
44.	Mrs Tigist Yeheyis	MoST
45.	Mr. Tewodros Wakuma	MoST
46.	Mr. Zelalem Dagne,	MoST
47.	Mr. Frew Hailu	MoST

This Guideline was produced with Technical and Financial support from the United States Presidents Emergency Plan for AIDS Relief (PEPFAR) through the U.S. Department of Health and Human Services, Centers for Disease Control and Prevention (HHS/CDC).

Contact Address:

NRERC Secretariat,

Ministry of Science and Technology,

Tel: +251118962583/4

nrerc2015@gmail.com,
